# Impact of the 2008 $M_W$ 7.9 Great Wenchuan earthquake on South China microplate motion

**DOI:** 10.1038/s41598-024-67141-3

**Published:** 2024-07-16

**Authors:** Giampiero Iaffaldano, Juan Martin de Blas, Xu Rui, D. Sarah Stamps, Zhao Bin

**Affiliations:** 1 Department of Chemistry, Life Science and Environmental Sustainability, University of Parma, Parma, Italy; 2 Department of Geosciences and Natural Resource Management, University of Copenhagen, Copenhagen, Denmark; 3 Institute for Disaster Management and Reconstruction, Sichuan University, Chengdu, China; 4 Department of Geosciences, Virginia Tech, Blacksburg, VA USA; 5 Institute of Seismology, China Earthquake Administration, Wuhan, China

**Keywords:** Natural hazards, Solid Earth sciences

## Abstract

Tectonic plate motions drive the earthquake cycle, as they result in the slow accrual and sudden release of energy along plate boundaries. Steadiness of plate motions over the earthquake cycle is a central tenet of the plate tectonics theory and has long been a main pillar in models of earthquake genesis, or of plate-margins seismic potential inferred from slip-deficit estimates. The advent of geodesy in the geosciences and the availability of multi-year-long series of position measurements permit tracking the motions of tectonic plates from before to after the time of significant seismic events that occur along their margins. Here, we present evidence that large earthquakes are capable of modifying the motions of entire microplates. We use high precision Global Navigation Satellite System (GNSS) position time-series covering the periods 2001–2004 and 2014–2017 to demonstrate that, contrary to the tenet above, the South China microplate motion changed after the 2008 $M_W$ 7.9 Great Wenchuan earthquake. The GNSS data and associated uncertainties indicate a plate motion slowdown of up to 20% that is beyond the possible impact of data noise and is thus tectonically meaningful. We use quantitative models of torque balance to show that generating this kinematic change requires a force upon the South China microplate compatible with that imparted by the Great Wenchuan earthquake of 2008. The existence of a kinematic signal linked to the earthquake cycle that impacts an entire microplate might offer an additional, novel perspective to assessing the hazards of earthquake-prone tectonic regions.

## Introduction

The earthquake cycle is a process whereby relative motions between adjacent tectonic plates determine the slow, decade to century-long buildup of stress along tectonic plate margins followed by sudden stress release through earthquakes^1^. The great majority of large, destructive earthquakes occurring within Earth’s crust can be studied in this context. A central assumption in the geosciences is that the plate motions that fuel the earthquake cycle are insensitive to the temporal variations of crustal fault stresses associated with such a cycle. This assumption is the foundation of most numerical models of spontaneous earthquake generation that implement rate- and state-dependent frictional laws^2–5^, or models of the seismic potential of global plate margins that employ estimates of slip-deficit^6–11^. Geodetic observations, in particular the Global Navigation Satellite System (GNSS), provide the opportunity to measure contemporary plate motions^12,13^. The use of GNSS position time-series spanning periods shorter than the typical duration of earthquake cycles^1^ allows resolving plate motions at different moments during such cycles. Recent analyses of GNSS data in the Mediterranean and Andean settings^14–17^ indicate non-steadiness of plate motions during the earthquake cycle and thus call for testing the tenet above in other tectonic settings.

**Figure 1:**
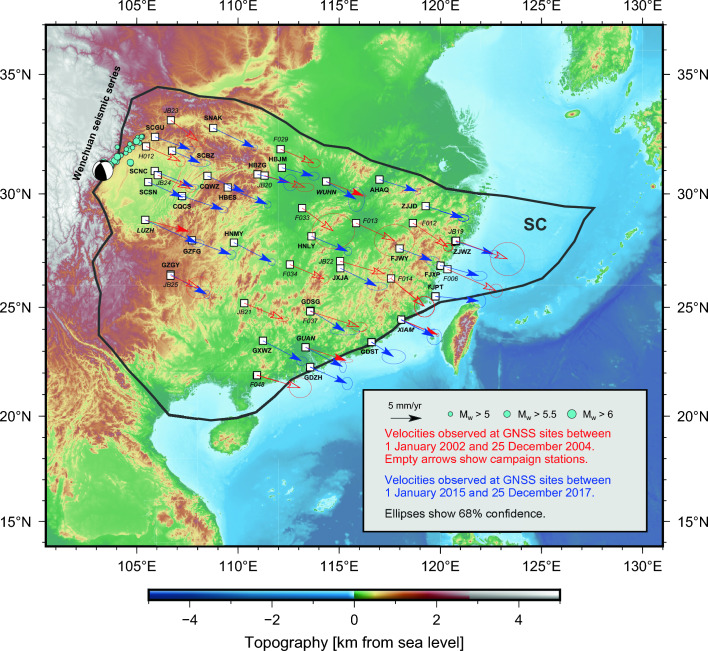
Velocities observed at continuously-recording (solid arrows) and campaign (empty arrows) GNSS stations used in this study to infer Euler vectors for the South China (SC, in black) microplate motion relative to Eurasia during the periods from January 2002 to December 2004 (in red), and from January 2015 to December 2017 (in blue). All GNSS stations are labeled: italics is used for campaign GNSS stations available during the earlier time period, bold for continuous GNSS stations, and bold-italics for the four continuously recording sites available for both the earlier and later time periods. The black beach ball shows the epicentre and USGS focal-mechanism solution of the 2008 M_W_7.9 Great Wenchuan earthquake. Turquoise dots are aftershocks. This figure was created utilising the Generic Mapping Tools version 6 (https://www.generic-mapping-tools.org/)^64^.

The South China (SC) microplate is an independently-moving continental lithospheric block located adjacent to the Eurasia plate, between the Tibetan Plateau and the trench where the Philippine Sea plate subducts beneath Eurasia^18^ (Fig. 1). The present-day SC motion is directed towards the East/Southeast relative to the Eurasian plate, as evidenced from contemporary GNSS measurements^19,20^, and is driven chiefly by the extrusion of Tibet^21^. Analyses of GNSS data^22^ indicate that most of SC is characterised by little internal deformation, with baseline linear strain-rate estimates around 1.5$\times 10^{-9}$ $yr^{-1}$. In contrast, the continental crust surrounding SC features strain-rates between 3 and 10 times larger^13^, which makes a precise identification of portions of the SC tectonic margins not straightforward^18,23^. On 12 May 2008, part of the western margin of SC ruptured with a $M_W$ 7.9 seismic event that remains one of the strongest recorded in China since the middle of the $19^{th}$ century and is referred to as the *Great Wenchuan earthquake*. The earthquake occurred on the Longmen Shan Fault system and was the result of rupture on two oblique reverse segments of the system, the longest being $\sim$240 km^24^ (Fig. 1). Finite fault solutions constrained from teleseismic wave recordings^25–28^, as well as focal mechanism solutions^29–32^, indicate that coseismic slip on the $\sim$ $220^{\circ }$ striking, $\sim$ $30^{\circ }$ depth-dipping fault segments that ruptured was oriented with a $\sim$ $120^{\circ }$ rake. Such a mechanism is consistent with stresses associated with crustal material being displaced from the Tibetan Plateau towards the strong crust of the Sichuan Basin as the Indian plate collides with Eurasia^33^. This focal mechanism solution means that during the interseismic period preceding the 2008 event, SC and the fault segments-to-rupture experienced a force oriented East/Northeast that was later relaxed by the Great Wenchuan earthquake. This force had a significant component along the direction of SC motion^19,20^ relative to the Eurasian plate. The working hypothesis of this study hinges on the notion that the 2008 Great Wenchuan earthquake suddenly imparted a force to SC that was, to first degree, opposite to the force generated by interseismic stresses. Here we test the hypothesis that this results in a change of the entire SC microplate motion. We analyse GNSS position time-series collected at sites of the Crustal Movement Observation Network Of China (CMONOC)^22,34^ over two 3- and 3.5-year-long periods, before and after the 2008 Wenchuan earthquake. We calculate GNSS velocities that we use to constrain statistically significant temporal changes of the Euler vector for the SC microplate motion from before to after the 2008 earthquake. Lastly, we estimate the force required to generate the inferred kinematic change. We find that the estimated force is comparable with that imparted to SC by the 2008 Great Wenchuan earthquake coseismic stress drop.

## South China microplate motion

### GNSS data

The motion of SC can be expressed using Euler vectors^35^, which is an approach that assumes minimal internal plate deformation—smaller than the uncertainty of velocity measurements constraining the Euler vector. We constrain SC motion from GNSS sites belonging to the CMONOC^22,34^ that are located within the SC margins, in most cases at least 100 km from the plate boundary. The CMONOC was established in 1998 and significantly developed after 2010. Specifically, we focus on data recorded at the available GNSS sites over two time periods, one before and one after the 2008 Great Wenchuan earthquake. The time period before the event is from 1 January 2002 to 25 December 2004 (in the following referred to as *earlier period*), and the time period after the event is from 1 January 2015 to 25 December 2017 (in the following referred to as *later period*). The 3-year-long periods are selected using the following criteria: (i) they are longer than 2.5 years, which is generally considered the minimum standard to obtain tectonically-representative motions^36^. (ii) Following previous studies^14^, the later time period begins at least one Maxwell time-interval of the sub-SC asthenosphere (see “Methods” and Supplementary Information) after the earthquake. The occurrence of the 2008 coseismic stress drop means that a force acting upon SC (one that has grown during the interseismic phase over the area that later ruptured) is suddenly removed. This causes a readjustment of the motion of the SC lithosphere that, in turn, exerts a sudden stress at the lithosphere-asthenosphere interface. The asthenosphere responds to that in a viscoelastic fashion^1,37^—meaning that there is an immediate (after the 2008 earthquake), predominantly-elastic response that transitions through time into a predominantly-viscous response. The time it takes for this transition to occur is of the order of the Maxwell time interval. Previous studies^38,39^ have shown that the postseismic deformation in SC following the 2008 event is negligible. Nonetheless, we take a cautious approach by choosing a later time period that begins after one Maxwell time interval from the 2008 earthquake. This is to ensures that GNSS sites measure velocities at a time when most of the already negligible post-seismic elastic response of the viscoelastic asthenosphere beneath SC has elapsed, and asthenospheric flow is characterised by a purely-viscous component. In other words, we minimise the possibility that the GNSS sites record any of the post-seismic elastic part of the response^37^. (iii) By the same logic, the selection of earlier/later periods aims at minimising any possible post-seismic effects of other large earthquakes occurred around SC, such as the 1999 Chi-Chi, the 2004 Sumatra, and the 2011 Tohoku earthquakes. Several independent studies indicate that the postseismic-relaxation signals associated with these events over the earlier/later periods become negligible if one moves as far away from the epicenters as SC is^39–41^. The fact that de-trended position time-series (available in the Supplementary Information) do not exhibit monotonic trends confirms those inferences. (iv) Lastly, the basis of our working hypothesis is that microplate motions might be impacted by temporal stress variations associated with any seismic cycle that occurs along their margins and that precedes large earthquakes. This hypothesis implies the possibility that the SC motion recorded at GNSS sites is impacted by other stress variations along the SC margins that are not part of the 2008 Great Wenchuan earthquake cycle, but are associated with the interseismic phase preceding large earthquakes yet to occur. A previous study^42^ used synthetic models of plate dynamics to determine that while in principle the seismic cycle associated with any earthquake occurring along the margins of a microplate has an impact onto its motion, only the impact of large earthquakes is indeed sufficiently large to change the microplate motion by more than the typical uncertainty associated with GNSS inferences. This suggests that contemporary GNSS motions of plates should not be expected to remain consistent to their long-term (i.e., derived from paleomagnetic observations) counterpart, but rather experience short-term changes in response to seismic cycles going on along their margins. For the reasons above, we select the later period to be relatively close in time to the 2008 coseismic stress drop, even if position time-series recorded at sites of CMONOC extend up to today. Our choices for the GNSS observation time periods assumes that interseismic stress might grow substantially in the latest interseismic phase^43^. Thus, with our GNSS observation time period choices, we aim to minimise potential superimposed effects of ongoing interseismic stress variations caused by unknown seismic cycles that have not yet reached the coseismic phase.

During the earlier time period—from 1 January 2002 to 25 December 2004—4 continuously-recording GNSS stations belonging to CMONOC were deployed in SC. During the later period—from 1 January 2015 to 31 December 2017—there were 29 continuously-recording GNSS stations in SC—including the 4 continuous stations available for the earlier period. These stations represent the entirety of continuous GNSS data ever collected within SC over these periods. Given the marked difference in number of continuous GNSS stations available for the earlier and later periods, we elect to complement the earlier period with CMONOC campaign GNSS data. We consider CMONOC campaign sites that have been recording between 1999 and 2004, fit a linear model, and are located at least 100 km away from the SC tectonic margins to ensure they are not affected by plate boundary deformation^13^. For the earlier period, CMONOC provides 17 campaign GNSS sites that meet such criteria. We process position time-series from these sites over the earlier and later time periods (see “Methods”) to obtain velocities and associated uncertainties (Fig. 1). All position time-series and velocities are linked in the Additional information section.

**Figure 2:**
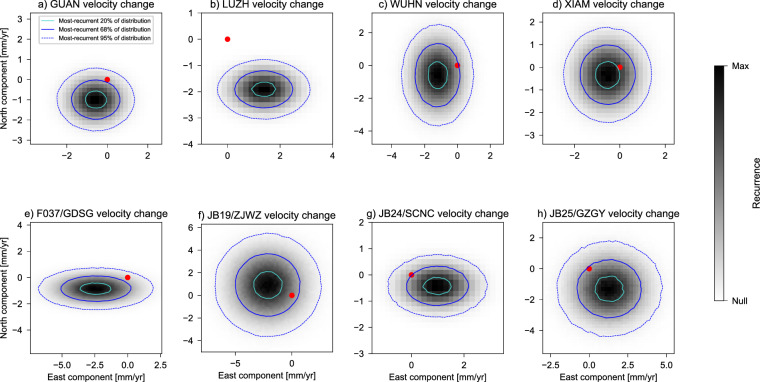
Distributions of velocity change at continuously-recording stations whose data cover both the earlier (from January 2002 to December 2004) and later (from January 2015 to December 2017) periods (**a**–**d**), as well as at pairs of closely-located (<30 km) sites (**e**–**h**). For the latter ones, data from the first site (campaign) in the label cover the earlier period, while data from the second site (continuously-recording) cover the later period. Distributions are obtained from the difference between ensembles of site velocities sampled from the nominal values and standard deviations of the East and North velocity components. Red dots show the origin (0,0), which would correspond to the centre of the distribution in case of no temporal change of site velocity. Contours show 20%, 68%, and 95% most-recurrent portions of the distributions. See Supplementary Information for maps of sites and site-pairs.

The continuous GNSS stations LUZH, GUAN, XIAM, and WUHN are common to both the earlier and later periods (see sites with full red and blue arrows in Fig. 1). In addition, 4 campaign sites (F037, JB19, JB24, and JB25) that recorded during the earlier period are located less than 30 km away from some of the continuous stations (GDSG, ZJWZ, SCNC, and GZGY) that recorded during the later period. Velocities at these sites provide a basis for a preliminary assessment of our hypothesis: we compare GNSS velocities observed during the earlier and later periods at the continuous stations LUZH, GUAN, XIAM, and WUHN, as well as at the 4 pairs of closely-located campaign/continuous sites (F037-GDSG, JB19-ZJWZ, JB24-SCNC, and JB25-GZGY)—see Supplementary Fig. 1 for the locations of these sites. We do so by generating ensembles of $10^6$ samples of East/North components of velocity using the nominal values and associated standard deviations. From these ensembles, we generate distributions of the temporal change of velocity components calculated by subtracting the ensemble of velocities during the earlier period from that of the later period (Fig. 2). Had there been no temporal change of velocity, the distributions would be centred at the origin—i.e., (0,0) in the 2D space of East/North velocity components. However, 5 out of 8 comparisons exhibit a slowdown of motion that is consistent with our hypothesis (Fig. 2). We note that the remaining 3 distributions, which exhibit microplate motion speedup, are systematically located in the part of SC featuring the longest Maxwell time-interval. This observation suggests that the velocities recorded at these sites during the later period (i.e., the one after the 2008 Wenchuan earthquake) may continue to be impacted by residual postseismic deformation. We note that the velocity comparison at site XIAM (Fig. 2d) does not support significant temporal change of motion at stricter levels of confidence, but we follow Gordon^35^ and test our hypothesis based on the analysis of SC Euler vectors, rather than any one single-site observation.

On the basis of the arguments above, we proceed as follows: we initially infer Euler vectors for SC microplate motion over the 3-year-long earlier and later periods starting from velocities from all available continuous GNSS stations. We compare Euler vectors and associated uncertainties, which propagate from standard deviations of site velocities. Next, we evaluate the robustness of any inferred temporal change using two independent statistical tests that weigh the number of observational constraints against the degrees of freedom of the inversion problem: the F-ratio and noise tests. Furthermore, we re-calculate Euler vectors for SC microplate motion also using velocities observed at continuous sites over 3.5-year long periods. Lastly, we repeat the Euler-vector inference in two additional cases: using position time-series from both continuously-recording and campaign GNSS sites over 3-year-long periods, as well as excluding from the analyses those sites located in the northwestern portion of SC featuring a higher Maxwell time-interval. Altogether, these analyses provide a basis for accepting or rejecting any evidence of temporal change with more confidence than that based on sole propagation of site-velocity uncertainties.

### Euler vectors for the South China microplate motion

**Figure 3:**
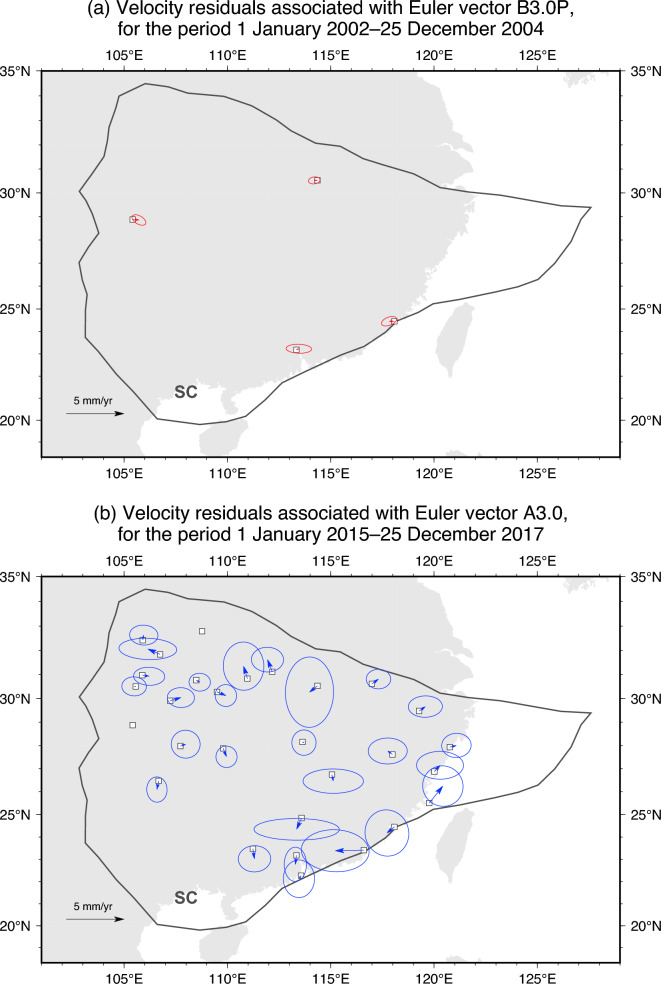
(**a**) Velocity residuals of GNSS stations (empty squares are stations positions) used to infer the SC/EU Euler vector labeled as B3.0P. (**b**) Same as (**a**), for the Euler vectors labeled as A3.0. Ellipses are 95% confidence. GNSS stations without residuals have been excluded from the Euler-vector inversion procedure (see main text for details). This figure was created utilising the Generic Mapping Tools version 6 (https://www.generic-mapping-tools.org/)^64^.

We constrain Euler vectors and associated covariances that represent the motion of SC relative to Eurasia before and after the Great Wenchuan earthquake as follows: for each time period, we start by generating $10^6$ samples of site velocities from the nominal solutions and their uncertainties. We then use these samples to solve the well-known inverse problem based on minimisation of the sum of squared misfits^44^ (see “Methods”), and obtain an ensemble of Euler vectors describing the SC motion. From these ensembles, we calculate mean values and covariances of the Cartesian components across the ensembles. After inverting for the best-fitting Euler vector, we check if all site velocity-residuals (i.e., the vectorial difference between observed site velocities and velocities predicted by the Euler vector at each site) are smaller than their 1-sigma uncertainties. If not, we iteratively repeat the inversion, each time removing, from the set of utilised sites, the one site yielding the largest unacceptable (i.e., larger than 2 standard deviations) residual in the previous iteration. The process stops when residuals of all utilised sites are acceptable. This approach is deliberate, and predicated on the following: realistically, the velocity sampled at each GNSS site is the SC motion, plus some (ideally negligible) contribution of other processes unrelated to the SC motion—whether geological in origin or not. The inversion scheme for Euler vectors weighs all utilised sites equally. On the one hand, the larger the number of utilised sites is, the more precise (i.e., smaller covariance) the Euler-vector inference is. On the other hand, if a specific site features a significant velocity-component unrelated to the SC motion, then such a component (i) impacts negatively onto the accuracy of the inferred Euler vector, and (ii) makes the velocity-residual of the specific site larger than the uncertainty. Our approach is to avoid the latter type of sites by iteratively removing them, and to to verify a-posteriori via noise tests (see below) that any undetected presence of non-plate-motion velocity-components in the pool of remaining sites is nonetheless innocuous to the working hypothesis. This approach carries a gain in terms of accuracy. The associated cost in terms of precision is in our opinion acceptable in light of the noise tests performed a-posteriori. In all cases related to the earlier period, residuals are acceptable when using all sites in the starting set of GNSS velocities, thus there is no need to remove any site in the Euler-vector inversion. In cases related to the later period, we iteratively remove up to 3 sites from the initial set of velocities (Table 1, Fig. 3 and Supplementary Information). The fact that no, or very few, sites need to be excluded in order to obtain acceptable residuals upon inversion for the best-fitting Euler vectors confirms a posteriori that the utilised networks of GNSS sites, indeed, record mostly the SC microplate motion.

**Figure 4:**
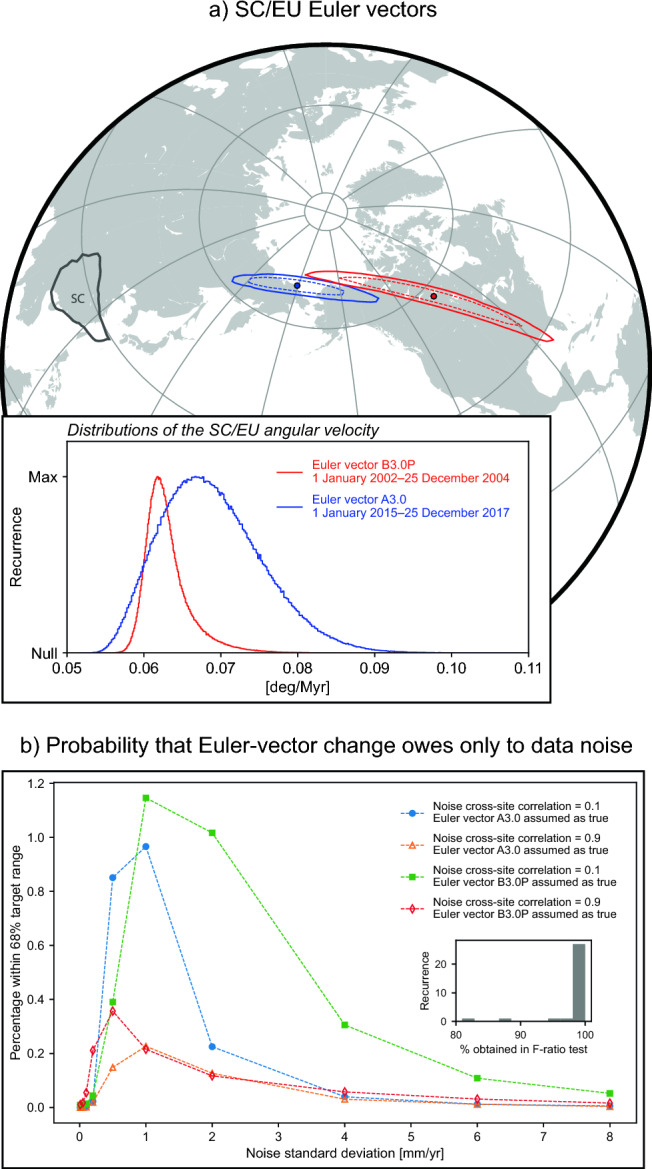
(**a**) Euler vectors for the motion of SC relative to EU before (B3.0P in Table 1) and after (A3.0 in Table 1) the 2008 Great Wenchuan earthquake, inferred from continuously-recording GNSS velocities (Fig. 1). Map shows the positions of the average Euler poles inferred for the two time periods, together with the contours where the most-recurrent 68% (dashed lines) and 95% (solid lines) of the sampled Euler poles fall. Continents are in light grey. SC margins are in dark grey. Inset shows the distributions of sampled angular velocities associated with the Euler poles. (**b**) Estimate of the probability that random, cross-site correlated noise in GNSS velocities is the only factor determining the difference between inferred Euler vectors for the earlier and later periods. Inset shows the histogram of the obtained F-ratio-test probabilities in noise tests (see main text for details). Part of this figure was created utilising the Generic Mapping Tools version 6 (https://www.generic-mapping-tools.org/)^64^.

Fig. 4A shows a comparison of the ensembles of Euler vectors obtained for the earlier and later 3-year-long periods when using only continuous GNSS stations. Similar figures illustrating results for the other cases explored are reported in the Supplementary Information. The distributions of Euler poles and angular velocities for the two time periods indicate a clear difference between the Euler vectors of the earlier period and the later period. The mean angular velocity for the later time period (in blue) falls outside the 68% confidence interval around the mean angular velocity of the earlier period (in red). Differences between distributions of earlier and later Euler vectors remain evident—although they do not necessarily follow the same patterns shown in Fig. 4A—also in the other cases explored (see Supplementary Information).

### Tests for the robustness of the inferred Euler-vector temporal change

Given two sets of GNSS velocities, the widely-employed F-ratio test^45^ assesses the probability that one single Euler vector would fit both sets to the same level or better than two distinct Euler vectors. We perform the F-ratio test on the pair of Euler vectors in Fig. 4A, and find there is less than 1.5% probability that a single Euler vector (i.e., one that has not changed through time) would fit the two sets of GNSS site-velocities as well as two distinct Euler vectors. To further test our hypothesis, we perform an additional set of statistical tests where we (i) assume—counter to our hypothesis—that there has been no temporal change of SC Euler vector, and (ii) assess the probability that the Euler-vector change evidenced by GNSS data is only apparent and owes to random, site-correlated data noise. Specifically, we perform semi-synthetic tests^14^ where we utilise computer-generated noise values and apply them to the SC motion inferred from actual GNSS velocities. In these tests, noise is intended as any site-velocity component that is unrelated to the SC microplate motion. Noise arises from other processes that may or may not be geological in origin.

We start by assuming that the SC Euler vector has not changed from before to after the 2008 earthquake, but has remained steady and equal to the Euler vector inferred from GNSS velocities for the period before the 2008 earthquake (labeled as B3.0P in Table 1). This assumption means that we assign to B3.0P the role of the true Euler vector and to the GNSS sites utilised for the period after the 2008 earthquake the role of noisy sites. By implication, the discrepancy between B3.0P and the Euler vector obtained for the post-Wenchuan period—A3.0 in this case—is assumed to be solely the result of data noise. Next, we use Euler vector B3.0P to calculate surface velocities at each of the GNSS sites used for the equally-long (i.e., 3 years) period after the 2008 earthquake. These are the velocities that GNSS sites would have recorded during the later period under the assumptions above and in the absence of any noise. We then add to each of these site velocities an ensemble of $10^6$ values of noise that have been randomly sampled from Gaussian distributions whose standard deviation is a free parameter in range from $10^{-2}$ to 8 mm/yr (referred to as *noise standard deviation*). Following previous studies^46^, we assume noise is correlated across sites and test correlation coefficient values of 0.1 and 0.9. For each value of noise standard deviation, we generate ensembles of $10^6$ noisy site velocities and calculate from these ensembles $10^6$ realisations of noisy Euler vectors. Next, we quantitatively assess how close the $10^6$ samples of noisy Euler vectors are to A3.0: we count what fraction of the ensemble of noisy Euler vectors (i.e., how many of the $10^6$ samples) falls inside a contour/interval (referred to as *target range*) where the most-recurrent 68% percent of the Euler pole/angular velocity of the A3.0 ensemble are located. Considering the size of the ensembles, such a fraction arguably represents a reasonable estimate of the probability that noise in the data utilised for the period after the 2008 earthquake is responsible for making the inferred Euler-vector appear different from that obtained using GNSS velocities for the time period before the earthquake. We repeat the same test, this time assigning the role of true Euler vector to A3.0, and the role of noisy sites to those utilised for the period before the 2008 earthquake.

Results show that such probability remains low (Fig. 4B, statistics associated with these tests in the other cases explored here are reported in the Supplementary Information). As part of these assessments (i.e., under the assumption that no actual, tectonically-meaningful change in SC microplate motion has occurred), we also perform F-ratio tests to sample the probability that, despite the presence of data noise, velocities observed before and after the 2008 earthquake can be fit by one single Euler vector (i.e., no temporal change of Euler vector) as well as by a set of two distinct Euler vectors. This serves to investigate the level of F-ratio-test probability that one can expect if there was no real change in the SC motion, and the only factor causing the inference of an apparent Euler-vector change was noise. The obtained probabilities exceed 85% (inset in Fig. 4B and Supplementary Information). These results suggest that it is unlikely that there has been no change in the SC motion following the 2008 Great Wenchuan event. The probability that (i) a single Euler vector is adequate to fit GNSS velocities recorded before and after the 2008 earthquake (i.e., that the whole-SC motion has not changed from before to after 2008), and (ii) the apparent change inferred when two distinct Euler vectors are fit to data (Fig. 4A) owes exclusively to data noise is the product of the probabilities obtained in the F-ratio and noise tests, which is at most 0.02% (Fig. 4B). Taken together, these statistical analyses support the notion of a real, tectonically-meaningful temporal change of the SC microplate Euler vector.

**Figure 5:**
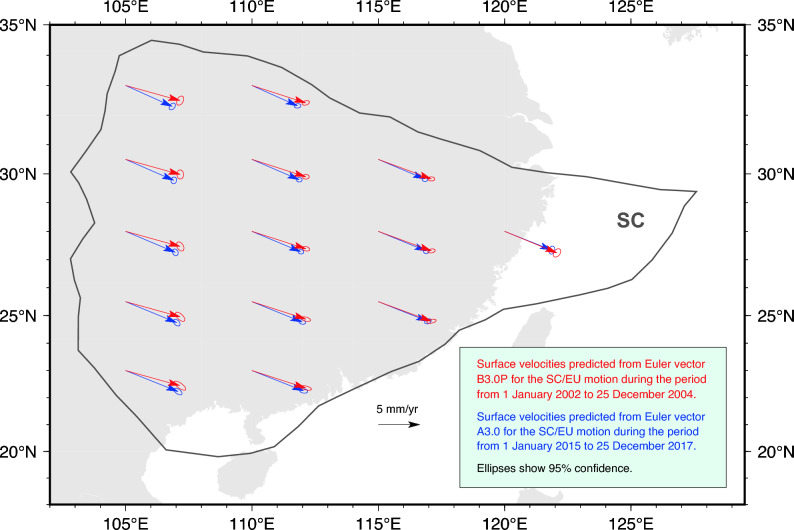
Surface velocities calculated at uniformly-spaced, arbitrary positions from the inferred Euler vectors: red arrows are calculated from B3.0P, blue arrows from A3.0 (see Table 1). Ellipses around velocity arrows show the 95% confidence. SC microplate margins are in black. Continents are in grey. This figure was created utilising the Generic Mapping Tools version 6 (https://www.generic-mapping-tools.org/)^64^.

Using the Euler vectors for the earlier and later periods, we calculate surface velocities at locations that are arbitrarily selected and uniformly spaced (Fig. 5). This illustrates how the Euler-vector temporal change inferred from GNSS data corresponds to a $\sim$20% slowdown of the East/Southeast-directed SC microplate motion. Surface velocities calculated from Euler vectors in the other cases explored here are reported in the Supplementary Information, and systematically illustrate a slowdown larger than that permitted by Euler-vector uncertainties.

## Torque-variations upon the South China microplate

**Figure 6:**
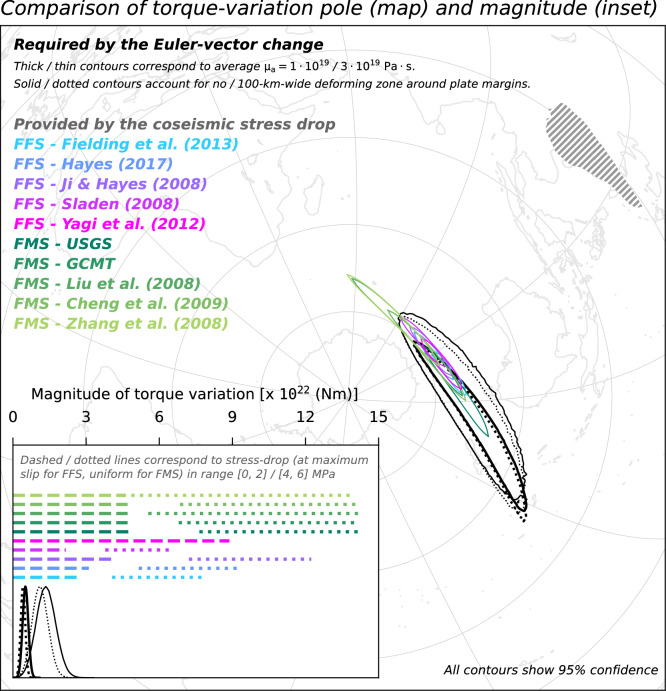
Comparison of the torque variation required upon SC to generate the plate-motion change evidenced by GNSS data (Euler vectors labeled as B3.0P and A3.0 in Table 1), and the torque variation provided to SC by the coseismic stress release of the 2008 Great Wenchuan earthquake. Acronyms FFS/FMS preceding the source reference stand for finite-fault/focal-mechanism solution, respectively. Map shows the regions where poles of the sampled torque-variation fall. Inset shows the distributions of the magnitude of the sampled torque-variations required upon SC, and compares them to ranges of torque-variation magnitudes provided by coseismic stress release. In hatched is the SC microplate boundary.

Any temporal change of the SC Euler vector must arise from a change in the torques acting upon SC. Parameterising the torque-balance equation for tectonic plates at two moments in time, and then taking their difference, yields a relationship that links changes in torque acting upon a plate to the associated change of the Euler vector via a linear operator (see “Methods”). The linear operator accounts for the geometry of the plate as well as the viscous resistance of the asthenosphere at its base^14,42,47^. We use the MYRIAM software^48^ to apply such parameterisation to the temporal change of the SC Euler vector in order to constrain the torque-variation needed upon SC to generate a 20% slowdown of its motion. Specifically, we utilise the ensembles of $10^6$ samples of the Euler vectors describing the motions of SC before and after the 2008 earthquake to generate ensembles of torque-variation vectors. Furthermore, we repeat such calculation in a second scenario, where we assume that the plate-tectonic approximation (i.e., the use of Euler vectors to describe the motion anywhere within a plate) progressively fails within 100 km of the SC margins (see “Methods”). This assumption means that crustal deformation is deemed non-negligible within such a 100-km-wide buffer zone around the SC margins. The distribution of the ensemble of torque-variation vectors can be visualised by focusing on the magnitude of the torque-variation and the geographical location where the torque-variation axis intersects Earth’s surface—also referred to as the torque-variation pole (Fig. 6). We sample the distribution of torque-variation required upon SC assuming radial and lateral heterogeneities in the viscosity of the asthenosphere. These heterogeneities are mapped from global surface-wave seismic tomography^49^ and scaled assuming end-member values for the global average viscosity of the asthenosphere equal to $1 \times 10^{19}$ and $3 \times 10^{19}$ Pa s ^50^ (see “Methods”).

**Figure 7:**
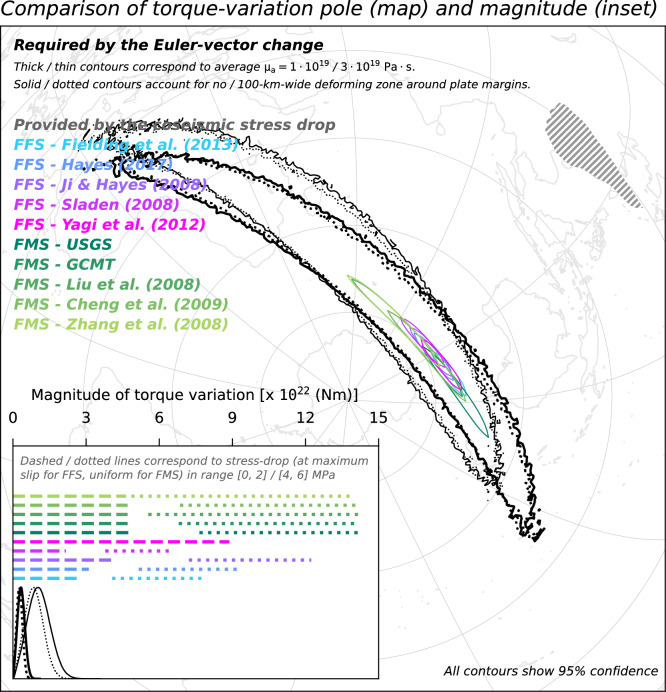
Same as Fig. 6, but for Euler vectors B3.0PC and A3.0 in Table 1.

**Figure 8:**
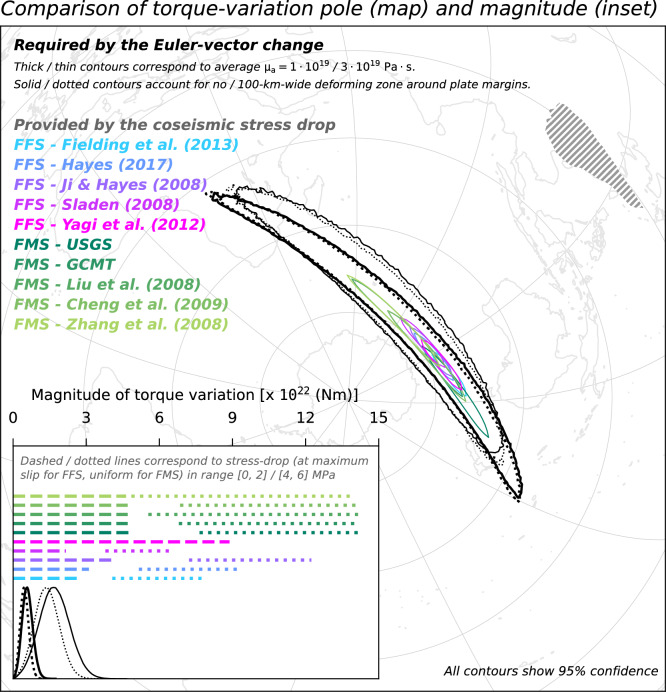
Same as Fig. 6, but for Euler vectors B3.0PC* and A3.0* in Table 1.

**Figure 9:**
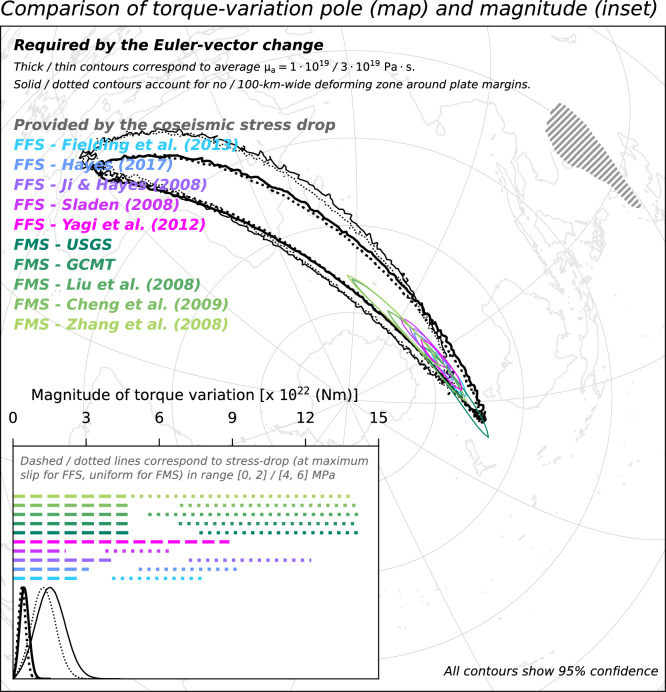
Same as Fig. 6, but for Euler vectors B3.5P and A3.5 in Table 1.

We compare the distribution of torque-variation required to act upon SC to generate the observed motion slowdown with the torque-variation imparted to SC by the coseismic stress drop of the Great Wenchuan earthquake. The latter torque-variation is calculated from models of either the finite-fault slip, which are constrained by teleseismic waves observations^25–28^, or of the focal mechanism^29–32^. In calculations relying on finite-fault solutions, we assume a linear relationship between coseismic slip and stress drop over the rupture area^51^. In calculations relying on focal-mechanism solutions, we assume a uniform stress drop over the rupture area (see “Methods”). These different approaches allow for testing the working hypothesis despite the large uncertainty on the actual value of the coseismic stress-drop and its lateral distribution over the rupture area. We test maximum stress-drop values in range from 0 to 2 and from 4 to 6 MPa, which are in line with global statistics^52^. Both the pole and magnitude of torque-variation required to act upon SC are compatible with what is imparted to SC by the rupture of the Great Wenchuan earthquake. This compatibility indicates that the force required to generate the observed slowdown of SC motion and the one provided by the 2008 earthquake are oriented in the same way. The compatibility also indicates that the two forces are of similar magnitude, regardless of the trade-offs between the controlling parameters that inevitably impact the torque calculations. These inferences also hold when using a hybrid set of campaign and permanent sites for calculating the Euler vector for the earlier period (Fig. 7); when excluding from the Euler-vector inference GNSS sites located in the SC region featuring a high Maxwell time-interval (Fig. 8); when using Euler vectors inferred from GNSS velocities calculated over a 3.5-year-long period (Fig. 9); as well as when accounting for possible non-negligible deformation near the SC margins (dotted contours/lines in Figs. 6, 7, 8, 9). The existence of a whole-plate kinematic signal associated with the occurrence of large earthquakes, combined with the ever-growing body of data provided by the network of continuously-recording GNSS sites^53^ might provide an additional perspective to efforts aimed at assessing the seismic potential of tectonic settings.

## Methods

Some of the methods presented below are reprised from previous studies (see references). They are reported here for completeness and reproducibility.

### Viscosity and Maxwell time interval of the asthenosphere

We resort to previous models of long-wavelength glacial rebound data^54,55^ that constrain the relationship between viscosity and thickness of the asthenosphere. Specifically, they found that the cube of the asthenosphere thickness ($H_a$) is proportional to the ratio of asthenosphere viscosity ($\mu _a$) over viscosity of the lower part of the upper mantle ($\mu _M$). That is, $H_a = a \cdot (\mu _a/\mu _M)^{1/3}$, with $a = 4.73 \cdot 10^5~\text{m}$. Setting the global average of $\mu _a$ to $5 \cdot 10^{19}$ Pa s and that of $\mu _M$ to $1.5 \cdot 10^{21}$  Pa s yields $H_a \sim 150~$km. Instead, $H_a \sim 90~$km when the global average of $\mu _a$ is set to $1 \times 10^{19}$ Pa s. Furthermore, we implement asthenosphere lateral variations of viscosity in line with the lateral temperature variations mapped by the tomography model PM_v2_2012 by Priestly and McKenzie^49^ (see Table 1 and Eqs. (1), (15), and (17) in their study). We also utilize PM_v2_2012 to map temperature-dependent lateral variations of the asthenosphere Young’s modulus, whose global average within the asthenosphere is set to $1.5 \times 10^{11}~$Pa^56^. Asthenosphere viscosity and Young’s modulus are used to infer lateral variations of the characteristic Maxwell time-interval of the asthenosphere. See Supplementary Information for figures illustrating the vertically-averaged asthenosphere viscosity, Young’s modulus, and Maxwell time-interval underneath the South China microplate.

**Table 1:** Euler vectors ($\mathbf {\omega }$) and associated covariances ($C_{ij}$) for the SC motion relative to Eurasia before and after the 2008 Great Wenchuan earthquake.

Label	lon	lat	Ang. velocity	$C_{xx}$	$C_{xy}$	$C_{xz}$	$C_{yy}$	$C_{yz}$	$C_{zz}$	Excluded sites
	° *E*	° *N*	[10^−2^ deg Myr^−2^]	[10^−8^ rad^2^ Myr^−2^]						
B3.0P	− 104.42	57.02	6.116	1	− 2	− 1	5	3	2	None
B3.5P	− 139.98	66.06	6.787	3	− 7	− 4	18	10	6	None
B3.0PC	− 134.26	65.90	6.356	1	− 3	− 2	8	5	3	None
B3.0PC*	− 111.94	57.56	6.353	4	− 10	− 5	22	12	7	None
A3.0	179.18	69.78	6.823	0	− 1	− 1	3	2	1	LUZH, SNAK
A3.0*	170.45	68.48	7.089	1	− 2	− 1	5	3	2	SNAK
A3.5	− 176.74	72.26	7.015	0	− 1	0	1	1	1	LUZH, HBJM, GDST

Labels are as follows: B/A refers to the time period before/after the earthquake. The number following B/A indicates the length in years of the position time-series used to calculate GNSS site velocities. A * appended to the label indicates use of the reduced network (see main text for further details).

### GNSS data processing

The GNSS data we processed are from the Crustal Movement Observation Network of China (CMONOC), operated by the China Earthquake Administration. We processed the CMONOC data together with data from the surrounding International GNSS Service (IGS) network to obtain loosely constrained daily solutions using the GAMIT/GLOBK software version 10.7^57^ (RELAX configuration), each with associated covariance matrices. We employed the latest antenna phase center models and IGS final satellite orbits in the International Terrestrial Reference Frame 2014 (ITRF2014) reference frame^58^. We utilized the empirical GPT2^59^ to determine a prior zenith tropospheric delay and Vienna Mapping Function 1 (VMF1) models for mapping the slant delays along each receiver-satellite path to the zenith. The local loosely constrained daily solutions and their covariances were then combined with global IGS solutions from Scripps Orbit and Permanent Array Center (SOPAC), and then fixed the combined daily solution into the 2014 International Terrestrial Reference Frame^58^. Initially, the combined time-series are edited: time-series points with sigma greater than 2 cm in either horizontal component or 4 cm in the vertical component are excluded preliminarily. Time-series points are deemed outliers and thus removed iteratively if the ratio of Res/(Sigma*NRMS) is greater than 2.5, where Res is the residual time-series point (after removing from the raw time-series a linear rate and offsets at breaks plus any user defined parameters), Sigma is the observed uncertainty of the time-series point, NRMS is the normalised root-mean-square of the residual time-series. The cleaned time-series are then used to derive site velocities in a Kalman filter estimator of trajectory models. Specifically, the trajectory models we employ include known discontinuities (resulting from instrument changes or other sources), a linear term (whose slope is the site velocity), as well as an annual term. Plots of the detrended time-series (i.e., observed series minus trajectory model) are reported in the Supplementary Repository associated with this study (see Additional Information section). For the later period, they show negligible postseismic deformation, in line with previous studies^38,39^. Realistic uncertainties of the velocities of the sites are obtained by imposing a random walk component to all continuous stations where the random walk process noise is set to generate the same velocity uncertainty as predicted by the first-order Gauss-Markov (FOGMEX) algorithm^60–62^. Velocities obtained for time periods before and after the Great Wenchuan earthquake are initially calculated in the ITRF2014 reference frame. To this end, we employ the glorg function of GAMIT/GLOBK. This generates a realisation of the reference frame based on globally distributed, stable GNSS stations. In our case, the number of stable stations utilised for deriving the reference frame falls within the range of 70 to 80, exceeding the minimum number of evenly distributed stations (40) suggested by statistical tests to ensure a stable solution. Sites close to the rupture zones of the megathrust subduction earthquakes, like the 2010 Mw=8.8 Maule, Chile and the 2011 Mw=9.0 Tohoku-oki, Japan earthquakes, are deliberately excluded from the set of sites. Previous studies have nonetheless already demonstrated that the later period used in this study does not include postseismic deformation^38,39^. Lastly, velocities obtained under the ITRF2014 framework are converted to the Eurasian fixed framework using the GAMIT/GLOBK utility cvframe, and are reported in the Supplementary Information.

### Inference of Euler vectors via minimisation of velocity squared misfits

Let $\textbf{r}_i$ ($i=1,2,...N$) be the position vectors of the sites within a tectonic plate where velocities are observed, let $\textbf{v}_{i}$ be the observed (i.e., measured) velocities at such sites, and let $\mathbf {\omega }$ be the Euler vector describing the plate motion. Lastly, let ${\hat{x}}$, ${\hat{y}}$, and ${\hat{z}}$ be the unit vectors of the *XYZ* Cartesian reference frame, in which any vector may be written. Therefore, for instance, $\mathbf {\omega }= \omega _x {\hat{x}} + \omega _y {\hat{y}} + \omega _z {\hat{z}}$. A theoretical prediction of site velocities associated with the plate motions is

1 $$\begin{aligned} \textbf{v}_{Ti} = \mathbf {\omega }\times \textbf{r}_i \end{aligned}$$

If the tectonic plate was moving perfectly rigidly over Earth’s surface, then theoretical and observed velocities would coincide – that is, $\textbf{v}_{Ti} = \textbf{v}_{i}$ for any value of the index *i*. Since observations are not perfect, one may infer $\mathbf {\omega }$ by requiring that a collective estimate of the difference between theoretical and observed velocities at the surface, also referred to as *velocity residual* $\textbf{R}_i = (\textbf{v}_{Ti} - \textbf{v}_i$), be minimum. Thus, one can first define the misfit function

2 $$\begin{aligned} M(\mathbf {\omega }) = \sum _{i=1}^N R_i^2 = \sum _{i=1}^N \bigg (\textbf{v}_{Ti} - \textbf{v}_{i} \bigg )^2 = \sum _{i=1}^N \bigg ((\mathbf {\omega }\times \textbf{r}_i) - \textbf{v}_{i} \bigg )^2 \end{aligned}$$

and then request that its gradient be zero, so that the misfit *M* is minimum. That is,

3 $$\begin{aligned} \frac{\delta M}{\delta \omega _x} = \frac{\delta M}{\delta \omega _y} = \frac{\delta M}{\delta \omega _z} = 0 \end{aligned}$$

Thus, it is

4 $$\begin{aligned} \frac{\delta M}{\delta \omega _x} = \frac{\delta }{\delta \omega _x} \sum _{i=1}^N \bigg ((\mathbf {\omega }\times \textbf{r}_i) - \textbf{v}_{i} \bigg )^2 = \sum _{i=1}^N \frac{\delta }{\delta \omega _x} \bigg [ (\omega _yr_{zi}-\omega _zr_{yi} - v_{xi})^2 + (\omega _zr_{xi}-\omega _xr_{zi} - v_{yi})^2 + (\omega _xr_{yi}-\omega _yr_{xi} - v_{zi})^2\bigg ] \end{aligned}$$

or

5 $$\begin{aligned} \begin{aligned} \frac{\delta M}{\delta \omega _x} = \sum _{i=1}^N \bigg [ 0 + 2(\omega _zr_{xi}-\omega _xr_{zi} - v_{yi})(-r_{zi}) + 2(\omega _xr_{yi}-\omega _yr_{xi} - v_{zi})(r_{yi}) \bigg ] = \\ = 2\sum _{i=1}^N \bigg [ \omega _x(r_{yi}^2+r_{zi}^2) + \omega _y(-r_{xi} r_{yi}) + \omega _z(-r_{xi} r_{zi}) - (v_{zi}r_{yi} - v_{yi}r_{zi}) \bigg ] \end{aligned} \end{aligned}$$

Therefore

6 $$\begin{aligned} \begin{aligned} \frac{\delta M}{\delta \omega _x} = \omega _x \Bigg [2\sum _{i=1}^N (r_{yi}^2+r_{zi}^2) \Bigg ] + \omega _y \Bigg [2\sum _{i=1}^N (-r_{xi} r_{yi}) \Bigg ] + \omega _z \Bigg [2\sum _{i=1}^N (-r_{xi} r_{zi}) \Bigg ] - \Bigg [2\sum _{i=1}^N (v_{zi}r_{yi} - v_{yi}r_{zi}) \Bigg ] \end{aligned} \end{aligned}$$

Similarly, it is

7 $$\begin{aligned} \begin{aligned} \frac{\delta M}{\delta \omega _y} = \omega _x \Bigg [2\sum _{i=1}^N (-r_{xi} r_{yi}) \Bigg ] + \omega _y \Bigg [2\sum _{i=1}^N (r_{xi}^2+r_{zi}^2) \Bigg ] + \omega _z \Bigg [2\sum _{i=1}^N (-r_{yi} r_{zi}) \Bigg ] - \Bigg [2\sum _{i=1}^N (v_{xi}r_{zi} - v_{zi}r_{xi}) \Bigg ] \end{aligned} \end{aligned}$$

and

8 $$\begin{aligned} \begin{aligned} \frac{\delta M}{\delta \omega _z} = \omega _x \Bigg [2\sum _{i=1}^N (-r_{xi} r_{zi}) \Bigg ] + \omega _y \Bigg [2\sum _{i=1}^N (-r_{yi} r_{zi}) \Bigg ] + \omega _z \Bigg [2\sum _{i=1}^N (r_{xi}^2+r_{yi}^2) \Bigg ] - \Bigg [2\sum _{i=1}^N (v_{yi}r_{xi} - v_{xi}r_{yi}) \Bigg ] \end{aligned} \end{aligned}$$

Posing the minimization condition in Eq. (3) therefore results in a linear system of three equations

9 $$\begin{aligned} {\left\{ \begin{array}{ll} \omega _x \cdot \sum _{i=1}^N (r_{yi}^2+r_{zi}^2) + \omega _y \cdot \sum _{i=1}^N (-r_{xi} r_{yi}) + \omega _z \cdot \sum _{i=1}^N (-r_{xi} r_{zi}) = \sum _{i=1}^N (v_{zi}r_{yi} - v_{yi}r_{zi}) \\ \omega _x \cdot \sum _{i=1}^N (-r_{xi} r_{yi}) + \omega _y \cdot \sum _{i=1}^N (r_{xi}^2+r_{zi}^2) + \omega _z \cdot \sum _{i=1}^N (-r_{yi} r_{zi}) = \sum _{i=1}^N (v_{xi}r_{zi} - v_{zi}r_{xi}) \\ \omega _x \cdot \sum _{i=1}^N (-r_{xi} r_{zi}) + \omega _y \cdot \sum _{i=1}^N (-r_{yi} r_{zi}) + \omega _z \cdot \sum _{i=1}^N (r_{xi}^2+r_{yi}^2) = \sum _{i=1}^N (v_{yi}r_{xi} - v_{xi}r_{yi}) \end{array}\right. }\ \end{aligned}$$

Alternatively, one can rename

10 $$\begin{aligned} \textbf{A} = { \begin{pmatrix} \sum _{i=1}^N (r_{yi}^2+r_{zi}^2) &{} \sum _{i=1}^N (-r_{xi} r_{yi}) &{} \sum _{i=1}^N (-r_{xi} r_{zi}) \\ \sum _{i=1}^N (-r_{xi} r_{yi}) &{} \sum _{i=1}^N (r_{xi}^2+r_{zi}^2) &{} \sum _{i=1}^N (-r_{yi} r_{zi}) \\ \sum _{i=1}^N (-r_{xi} r_{zi}) &{} \cdot \sum _{i=1}^N (-r_{yi} r_{zi}) &{} \sum _{i=1}^N (r_{xi}^2+r_{yi}^2) \end{pmatrix}}, \textbf{b} = { \begin{pmatrix} \sum _{i=1}^N (v_{zi}r_{yi} - v_{yi}r_{zi}) \\ \sum _{i=1}^N (v_{xi}r_{zi} - v_{zi}r_{xi}) \\ \sum _{i=1}^N (v_{yi}r_{xi} - v_{xi}r_{yi}) \end{pmatrix}} \end{aligned}$$

and express the minimization condition as

11 $$\begin{aligned} \textbf{A} \mathbf {\omega }= \textbf{b} \end{aligned}$$

This leads to inferring the Euler vector as

12 $$\begin{aligned} \mathbf {\omega }= \mathbf {A^{-1}} \textbf{b} \end{aligned}$$

where the right-hand side of equation (12) is known from observations. As observations $\textbf{v}_i$ typically come with uncertainties, one can first use these to generate an ensemble of $N_{\omega }$ samples of each $\textbf{v}_i$, and then apply the inversion scheme above to generate an equally large ensemble of $\mathbf {\omega }$. Realistic covariances associated with $\mathbf {\omega }$ can also be calculated from such ensemble, provided that $N_{\omega }$ is sufficiently large. In this study, we use $N_{\omega } = 10^6$ and assume no correlation between the East- and North-directed components of $\textbf{v}_i$. A set of Matlab functions that implement the inversion illustrated above is released as part of this study (see Supplementary Information). Table 1 reports Euler vectors and associated covariances inferred via the method above.

### Torque variation required to generate plate-motion changes

Previous studies^42,47^ obtained an analytical equation linking the torque variation $\Delta \textbf{M}$ experienced by a tectonic plate of basal area *S* to the resulting temporal change of Euler vector. Writing two equations that express the torque balance of a tectonic plate at two distinct points in time, $t_1$ and $t_2$, and then taking the difference between them, so that only the terms that have actually changed through time remain in the resulting equation, results in

13 $$\begin{aligned} \Delta \textbf{M} = \int _{S} \frac{\mu _a}{H_a} \cdot \textbf{r} \times [\textbf{v}_p(\textbf{r},t_2) - \textbf{v}_p(\textbf{r},t_1) ] \cdot dS \end{aligned}$$

where $\mu _a$ and $H_a$ are viscosity and thickness of the asthenosphere, $\textbf{v}_p(\textbf{r},t)$ is the plate motion at position $\textbf{r}$ and time *t*^42,47^, while *dS* is the infinitesimal element of plate basal surface *S* centred around position $\textbf{r}$. The plate-tectonic description means that $\textbf{v}_p(\textbf{r},t) = \mathbf {\omega }(t) \times \textbf{r}$, which would make Eq. (13) become

14 $$\begin{aligned} \Delta \textbf{M} = \int _{S} \frac{\mu _a}{H_a} \cdot \textbf{r} \times [\Delta \mathbf {\omega }\times \textbf{r}] \cdot dS \end{aligned}$$

where $\Delta \mathbf {\omega }= \mathbf {\omega }(t_2) - \mathbf {\omega }(t_1)$. The possibility that deformation near plate margins may be non-negligible would imply that surface velocities and their temporal changes near plate margins would not strictly follow the Euler-vector description. In order to account for this, one may introduce the option of locally dampening temporal kinematic changes implied by Euler-vector changes. To very first order, this notion can be implemented by means of a location-dependent dampening factor $D_{\textbf{r}} \le 1$. $D_{\textbf{r}} = 1$ if position $\textbf{r}$ is sufficiently away from plate margins, while $D_{\textbf{r}}$ decreases from 1 to 0 as $\textbf{r}$ gets closer to plate margins. Thus, Eq. (14) becomes

15 $$\begin{aligned} \Delta \textbf{M} = \int _{S} \frac{D_{\textbf{r}} \; \mu _a}{H_a} \cdot \textbf{r} \times [\Delta \mathbf {\omega }\times \textbf{r}] \cdot dS \end{aligned}$$

The fact that the Euler-vector change $\Delta \mathbf {\omega }$ is independent of $\textbf{r}$ means that the integral may be written as the result of a linear map

16 $$\begin{aligned} \int _{S} \frac{D_{\textbf{r}} \; \mu _a}{H_a} \cdot \textbf{r} \times [\Delta \mathbf {\omega }\times \textbf{r}] \cdot dS = \underbrace{ \begin{pmatrix} \int _{S} \frac{D_{\textbf{r}}\mu _a}{H_a}(y^2+z^2)dS &{} -\int _{S} \frac{D_{\textbf{r}}\mu _a}{H_a}xydS &{} -\int _{S} \frac{D_{\textbf{r}}\mu _a}{H_a}xzdS \\ -\int _{S} \frac{D_{\textbf{r}}\mu _a}{H_a}xydS &{} \int _{S} \frac{D_{\textbf{r}}\mu _a}{H_a}(x^2+z^2)dS &{} -\int _{S} \frac{D_{\textbf{r}}\mu _a}{H_a}yzdS \\ -\int _{S} \frac{D_{\textbf{r}}\mu _a}{H_a}xzdS &{} -\int _{S} \frac{D_{\textbf{r}}\mu _a}{H_a}yzdS &{} \int _{S} \frac{D_{\textbf{r}}\mu _a}{H_a}(x^2+y^2)dS \end{pmatrix}}_{\textbf{P}} {\begin{pmatrix} \Delta \omega _x \\ \Delta \omega _y \\ \Delta \omega _z \end{pmatrix}} \end{aligned}$$

where the operator $\textbf{P}$ links the vectorial space of Euler-vector changes that a tectonic plate could experience to that of torque variations possibly acting upon it. Therefore,

17 $$\begin{aligned} \Delta \textbf{M} = \textbf{P} \Delta \mathbf {\omega }\end{aligned}$$

Equation (17) holds when the time periods over which $\Delta \textbf{M}$ and $\Delta \mathbf {\omega }$ are accomplished are (i) short enough (in a geological sense) that the plate has not changed shape, but also (ii) long enough that the elastic component of sublithospheric motion within the viscoelastic asthenosphere—which affects velocities of the overlying plate^37^—has decayed to a significant extent. These conditions indeed apply to SC for Euler vectors calculated using GNSS data collected between 2001 and 2004, and between 2014 and 2017, since these two periods are more than 2–3 Maxwell time-intervals apart from each other (see Supplementary Information). We draw $10^6$ samples of the SC Euler vectors (Table 1) for periods before and after the Great Wenchuan earthquake, and use them to build ensembles of geodetically-constrained SC Euler-vector changes that we then map, through equation (17), into ensembles of torque variations needed to generate the SC temporal change of motion. In doing so, we implement the findings of previous studies of long-wavelength glacial rebound data^54,55^, which constrain the cube of the asthenosphere thickness to be proportional to the viscosity contrast between the asthenosphere and the upper mantle. In addition, we also implement asthenosphere lateral variations of viscosity in line with the lateral temperature variations mapped by the tomography model PM_v2_2012^49^. Furthermore, we explore the impact of two distinct scenarios for the dampening factor $D_{\textbf{r}}$: in one scenario, we set $D_{\textbf{r}} = 1$ everywhere inside *S*. This means assuming SC as perfectly rigid. In the second scenario, we assume a 100-km-wide internal buffer zone along the SC margins where deformation is non-negligible. Inside this zone, $D_{\textbf{r}}$ decreases linearly from 1 to 0 as $\textbf{r}$ gets closer to the SC margins, and becomes equal to 0 on the SC margins. The entries of the SC-specific operators $\textbf{P}$ when the viscosity of the upper mantle is set to $1.5 \cdot 10^{21}$ Pa s and $\mu _a$ is set variably to $1 \cdot 10^{19}$ and $3 \times 10^{19}$ Pa s^50,63^ are reported in the Supplementary Information. We visualise the distribution of $\Delta \textbf{M}$ by plotting the histogram of its magnitude $| \Delta \textbf{M} |$ and the contour containing 95% of the geographical positions where the sampled torque-variation directions $\Delta \textbf{M}/ | \Delta \textbf{M} |$ intersect Earth’s surface—also referred to as torque-variation poles. All the above is performed via the MYRIAM open-source software^48^.

### Torque variation from co-seismic stress drop

Following previous studies, we calculate the torque variation imparted to SC by the 2008 Great Wenchuan earthquake rupture ($\Delta \textbf{M}_e$) using two methods, one utilising finite-fault slip solutions, the other utilising focal-mechanism solutions. In the former case, we follow the method of Martin de Blas et al. (2022)^14^ and proceed by integrating the coseismic stress drop occurred during the earthquake over the finite rupture area. We assume that the stress drop over the rupture area scales linearly with the amount of coseismic slip^51^, which has been estimated through teleseismic wave observations^25–28^ and is available in the form of finite-fault rupture solutions at http://equake-rc.info/srcmod/^27^. In quantitative terms, we implement the following equation

18 $$\begin{aligned} \Delta \textbf{M}_e = \int _{\Sigma _r} \textbf{r} \times \bigg [ \Delta \sigma _{max} \cdot \frac{s(\textbf{r})}{s_{max}} \cdot {\hat{u}}(\textbf{r}) \bigg ] \cdot d\Sigma _r \end{aligned}$$

where $\Delta \sigma _{max}$ is the maximum stress drop over the rupture area $\Sigma _r$ (which is defined by the strike and dip of the specific finite-fault rupture solution), $s(\textbf{r})$ is the amount of slip at position $\textbf{r}$ over $\Sigma _r$, $s_{max}$ is the maximum slip over the rupture area, while ${\hat{u}}(\textbf{r})$ is direction of rake at position $\textbf{r}$. For each of the finite-fault solutions utilised here^25–28^, we generate $10^5$ samples of $\Delta \textbf{M}_e$. In doing so, we vary randomly the strike of the surface projection of the rupture by $\pm 3^{\circ }$ (corresponding to a relocation of the surface projection by up to 5 km). We also vary the dip of the fault and the slip rake prescribed by the specific finite-fault solution by $\pm 5^{\circ }$. We keep $\Delta \sigma _{max}$, occurring where slip is maximum, as a free parameter that we vary uniformly in range from 0 to 2 MPa, and from 4 to 6 MPa—in line with global statistics of strike-slip earthquakes^52^. We randomly select values of the controlling parameters within the ranges/sets above, and integrate the infinitesimal torque associated with the infinitesimal stress-drop vector over the rupture area surface. This allows us to generate an ensemble of $10^5$ samples of torque variations $\Delta \textbf{M}_e$ imparted to SC by the Great Wenchuan earthquake rupture.

For calculations of $\Delta \textbf{M}_e$ that rely on focal-mechanism solutions, we implement the method of Iaffaldano et al. (2022)^15^: the direction of the coseismic stress associated with the occurrence of an earthquake – here indicated with the unit-vector symbol ${\hat{s}}$ – can be determined from the strike, dip, and rake (the latter one is a scalar values in the case of focal-mechanism solutions) of the main nodal plane, while its magnitude $\Delta \sigma$ is kept as a free parameter. The torque variation $\Delta \textbf{M}_e$ is then parameterised as

19 $$\begin{aligned} \Delta \textbf{M}_e = (\Delta \sigma \cdot \Sigma _e) \cdot [\textbf{r}_e \times {\hat{s}}] \end{aligned}$$

where $\textbf{r}_e$ is the position of the earthquake hypocentre, while $\Sigma _e$ is the size of the rupture area. We constrain the unit vector ${\hat{s}}$ from various focal mechanism solutions – i.e., strike, dip, and rake – available in the scientific literature. We elect to assume an uncertainty on each of the parameters equal to $7^{\circ }$, which is in line with statistical analyses of the focal mechanism parameters of the 2008 Great Wenchuan earthquake^30^. As in the case of calculations relying on finite-fault slip solutions, we take the stress drop $\Delta \sigma$ variably in range from 0 to 2 MPa, and from 4 to 6 MPa. We draw $10^5$ samples of these parameters from uniform distributions within the ranges indicated above, and build an ensemble of $10^5$ realisations of $\Delta \textbf{M}_e$. We visualise the distribution of $\Delta \textbf{M}_e$ by plotting the range of sampled magnitudes, assuming two intervals of possible values for $\Delta \sigma _{max}$, and the contour containing 99% of the geographical positions where the sampled torque-variation directions $\Delta \textbf{M}_e/ | \Delta \textbf{M}_e |$ intersect Earth’s surface – also referred to as torque-variation poles.

### Supplementary Information

Supplementary Information 1.

Supplementary Information 2.

## Data Availability

All data generated or analysed during this study are either included in this published article (and its Supplementary information files) or available at https://zenodo.org/records/12518003.
