# Supplementary material for: Impact of the 2008 MW 7.9 Great Wenchuan earthquake on South China microplate motion
Source: Sci Rep. 2024 Jul 16;14:16469. doi: 10.1038/s41598-024-67141-3 (PMC11252374; doi:10.1038/s41598-024-67141-3)
Supplement: Supplementary file 1 — Supplementary Information 1. [file 41598_2024_67141_MOESM1_ESM.pdf]

# Impact of the 2008 $M_W$ 7.9 Great Wenchuan earthquake on South China microplate motion

Giampiero Iaffaldano[a,*], Juan Martin de Blas[b], Xu Rui[c,*], D. Sarah Stamps[d], Zhao Bin[e]

[a]*Department of Chemistry, Life Science and Environmental Sustainability, University of Parma, Italy.*
[b]*Department of Geosciences and Natural Resource Management, University of Copenhagen, Denmark.*
[c]*Institute for Disaster Management and Reconstruction, Sichuan University, Chengdu, China*
[d]*Department of Geosciences, Virginia Tech, Blacksburg, Virginia, USA*
[e]*Institute of Seismology, China Earthquake Administration, Wuhan, China.*

# Supplementary information

## Supplementary files list

**Supplementary File 1**. Velocities of continuously–recording GNSS stations utilised to constrain SC Euler vectors relative to Eurasia for the period from January 2002 to December 2004. Columns are: (1) sites acronyms, (2-3) longitude (decimal deg East) and latitude (decimal deg North) of site locations, (4-5) station East/North velocities (mm/yr) relative to Eurasia, and (6-7) standard deviations of the East/North velocities (mm/yr).

**Supplementary File 2**. Same as Supplementary File 1, but for the period from July 2001 to December 2004.

**Supplementary File 3**. Same as Supplementary File 1, but for campaign GNSS stations recording during the period from January 1999 to December 2004.

**Supplementary File 4**. Same as Supplementary File 1, but for the period from January 2015 to December 2017.

*Corresponding authors
*Email addresses:* giia@ign.ku.dk (Giampiero Iaffaldano), xurui_3@163.com (Xu Rui)

**Supplementary File 5**. Same as Supplementary File 1, but for the period from July 2014 to December 2017.

**Supplementary File 6**. Software for Euler vector inversion (see Methods).

## Supplementary tables

Supplementary Table 1: Entries of the linear operator **P** (see Methods). Units are $10^{38}$ $\mathrm{Pa \cdot s \cdot m^3}$. Label *non-rigid* means that the operator accounts for a 100-km-wide deforming zone internal to SC, located around its margins.

| CASE | $\mathbf{P}_{11}$ | $\mathbf{P}_{12}$ | $\mathbf{P}_{13}$ | $\mathbf{P}_{22}$ | $\mathbf{P}_{23}$ | $\mathbf{P}_{33}$ |
|---|---|---|---|---|---|---|
| $\mu_a = 1e19$ Pa · s, rigid | 10.9650 | 2.7683 | 1.6416 | 3.9526 | -4.8086 | 9.1305 |
| $\mu_a = 3e19$ Pa · s, rigid | 34.1020 | 10.1480 | 5.8980 | 13.1540 | -14.7490 | 29.8830 |
| $\mu_a = 1e19$ Pa · s, non-rigid | 8.8507 | 2.2342 | 1.3181 | 3.1535 | -3.8710 | 7.3745 |
| $\mu_a = 3e19$ Pa · s, non-rigid | 28.2230 | 8.3346 | 4.8094 | 10.6930 | -12.1670 | 24.7030 |

## Supplementary figures

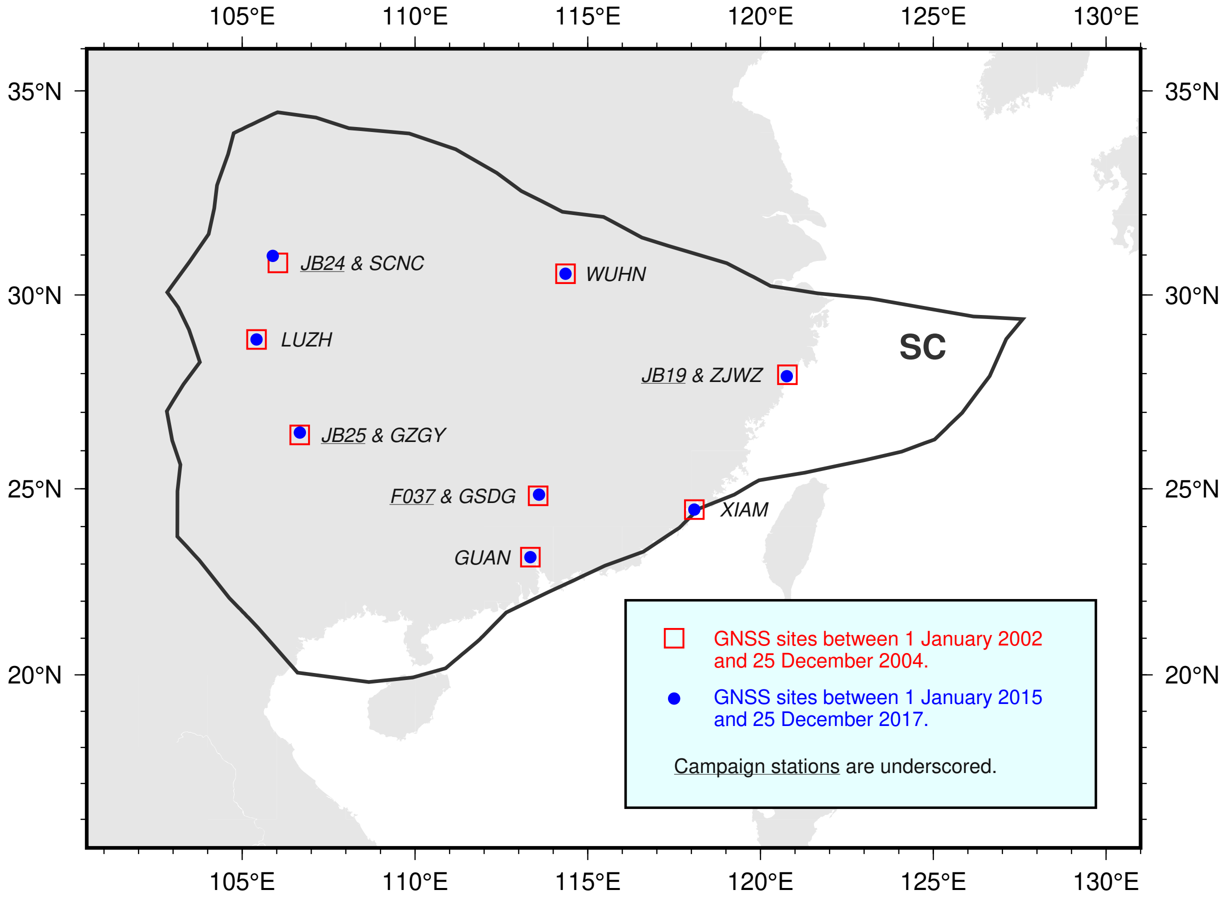


Supplementary Figure 1: Locations of GNSS stations and station–pairs utilised to generate distributions of station velocity–changes (see Fig. 2 in the main text). In red are stations whose data cover the earlier time period (underscored station names indicate a campaign station, others are continuously–recording stations), in blue those whose data cover the later time period. Data from stations GUAN, LUZH, WUHN, and XIAM cover both the earlier and later periods.

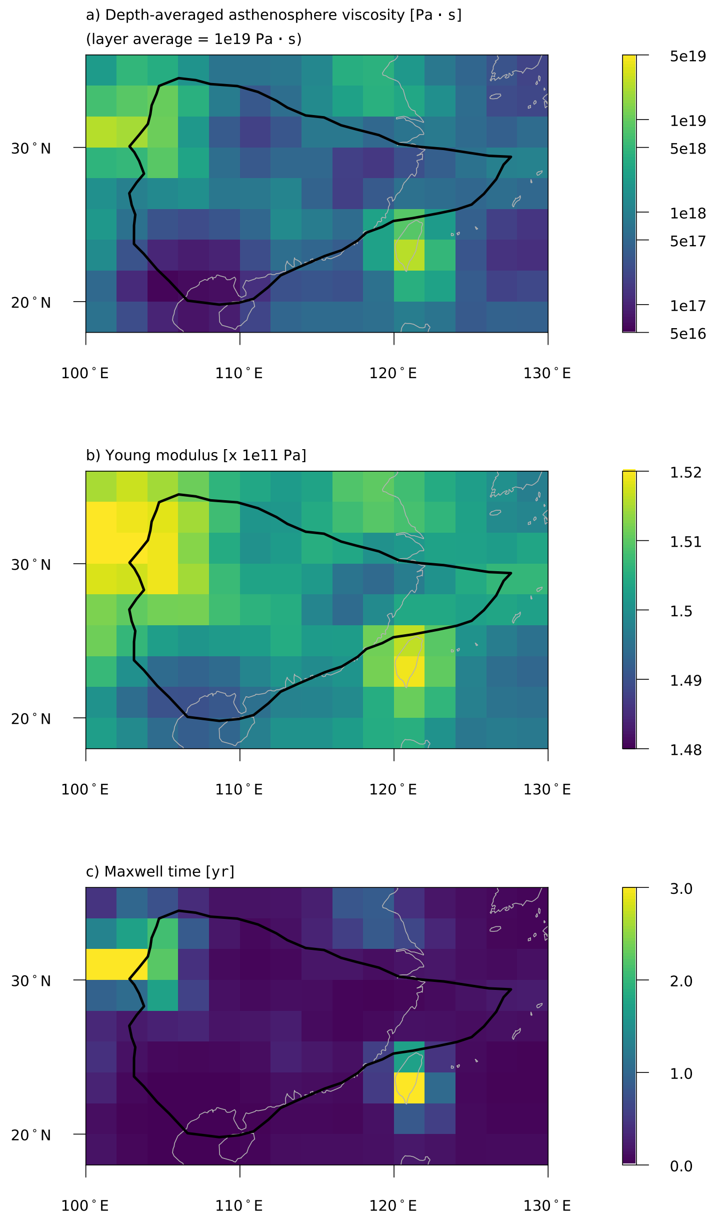


Supplementary Figure 2: Depth–averaged asthenosphere viscosity (a), Young's modulus (b), and Maxwell time–interval (c) underneath the South China microplate, calculated assuming the global average of the asthenosphere viscosity $\mu_a$ to be $1 \cdot 10^{19}$ Pa · s. Lateral variations of $\mu_a$ are inferred from lateral temperature variations in the seismic tomography model of Priestley & McKenzie (2013). South China microplate margins are in thick black. Coastline is in gray.

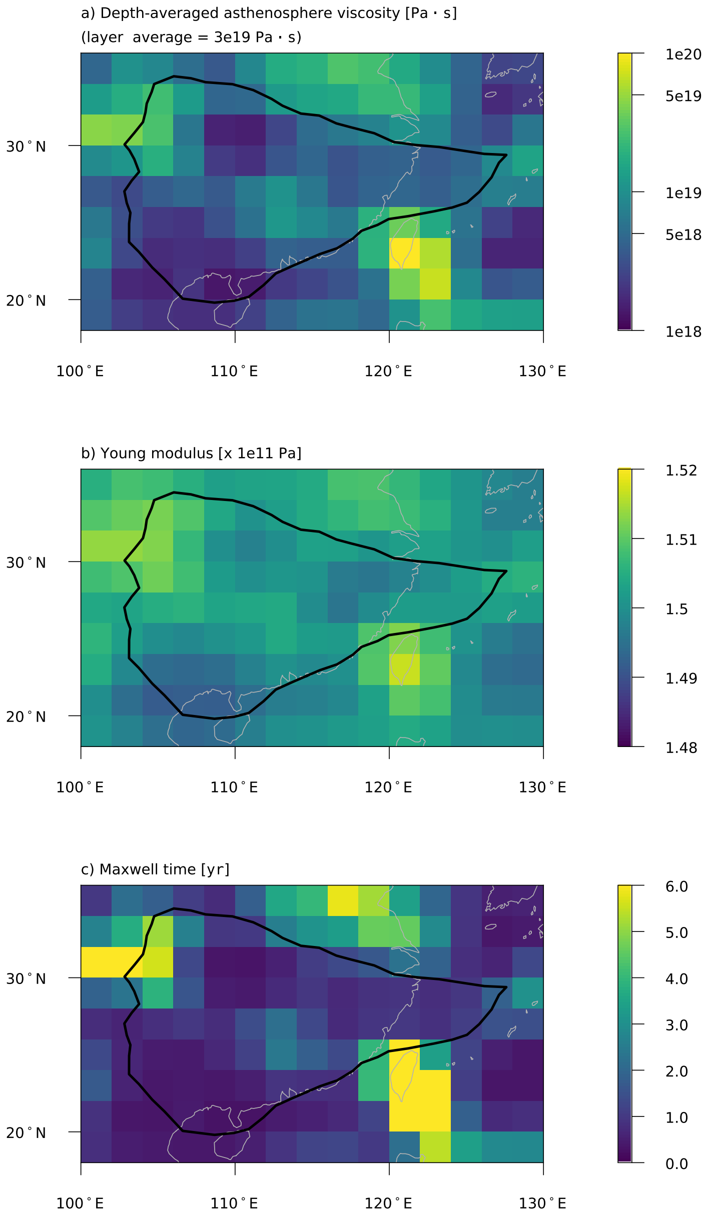


Supplementary Figure 3: Same as Supplementary Figure 2, but for $\mu_a = 3 \cdot 10^{19}$ Pa · s.

(a) Velocity residuals associated with Euler vector B3.0PC, for the period 1 January 2002–25 December 2004

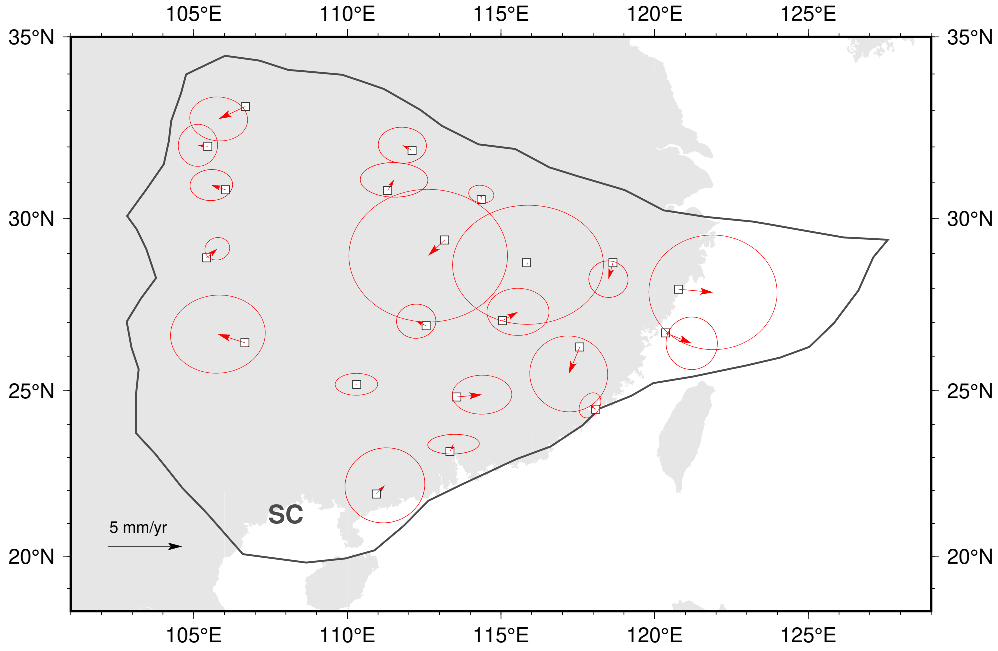


(b) Velocity residuals associated with Euler vector A3.0, for the period 1 January 2015–25 December 2017

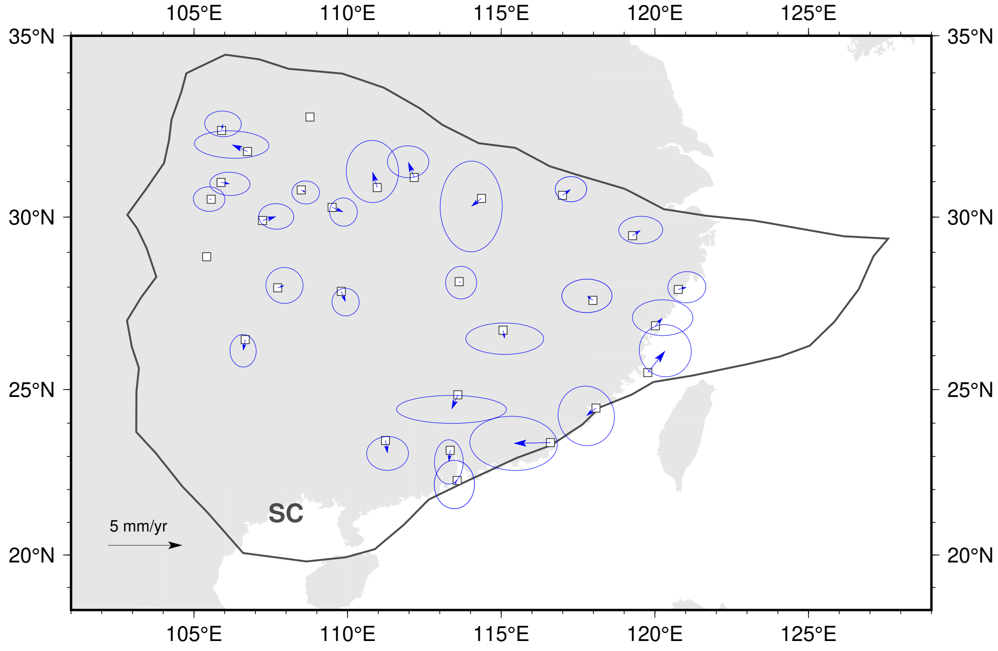


Supplementary Figure 4: Same as Fig. 3 in the main text, but for SC/EU Euler vector B3.0PC (panel a) and A3.0 (panel b) in Table 1 of the main text.

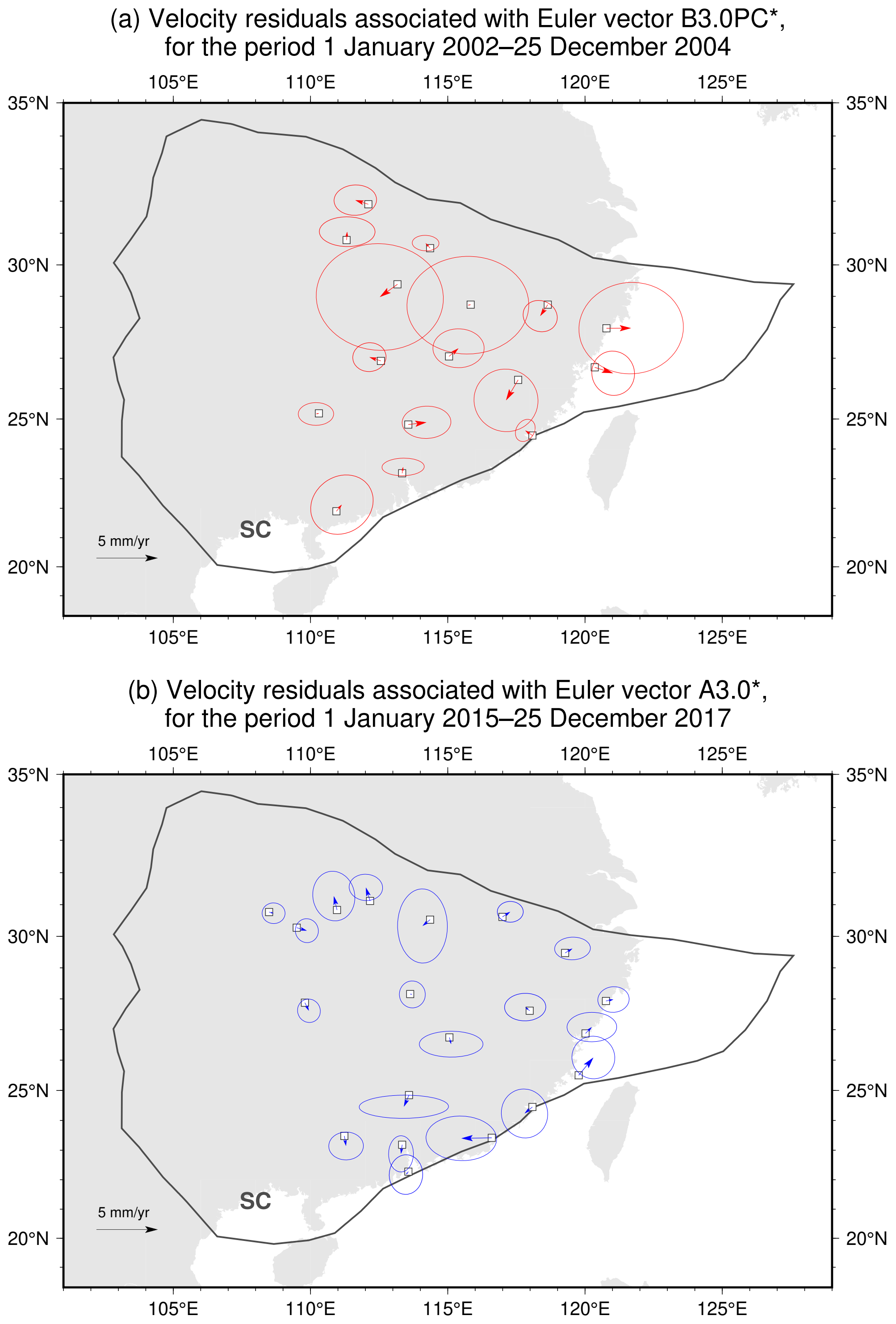


Supplementary Figure 5: Same as Fig. 3 in the main text, but for SC/EU Euler vector B3.0PC* (panel a) and A3.0* (panel b) in Table 1 of the main text.

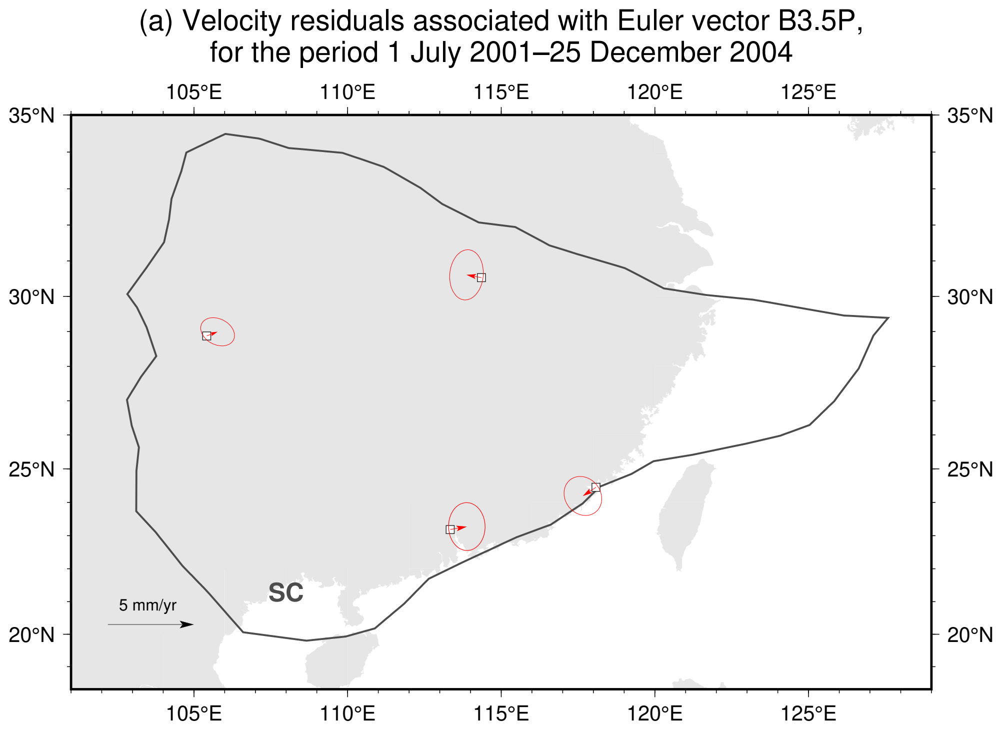


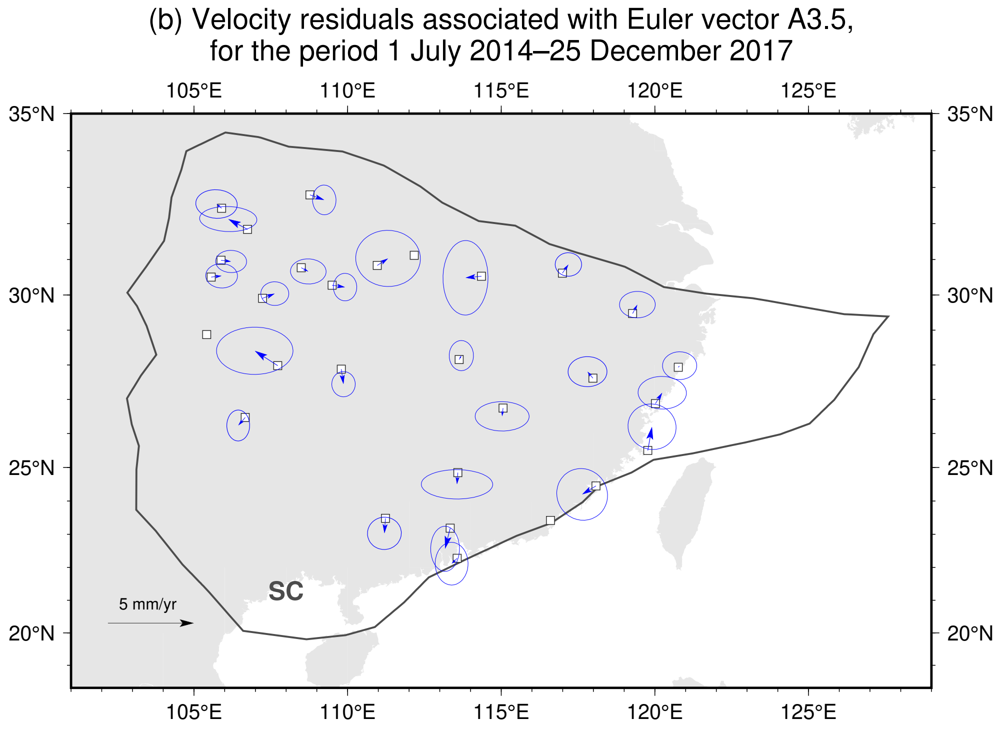


Supplementary Figure 6: Same as Fig. 3 in the main text, but for SC/EU Euler vector B3.5P (panel a) and A3.5 (panel b) in Table 1 of the main text.

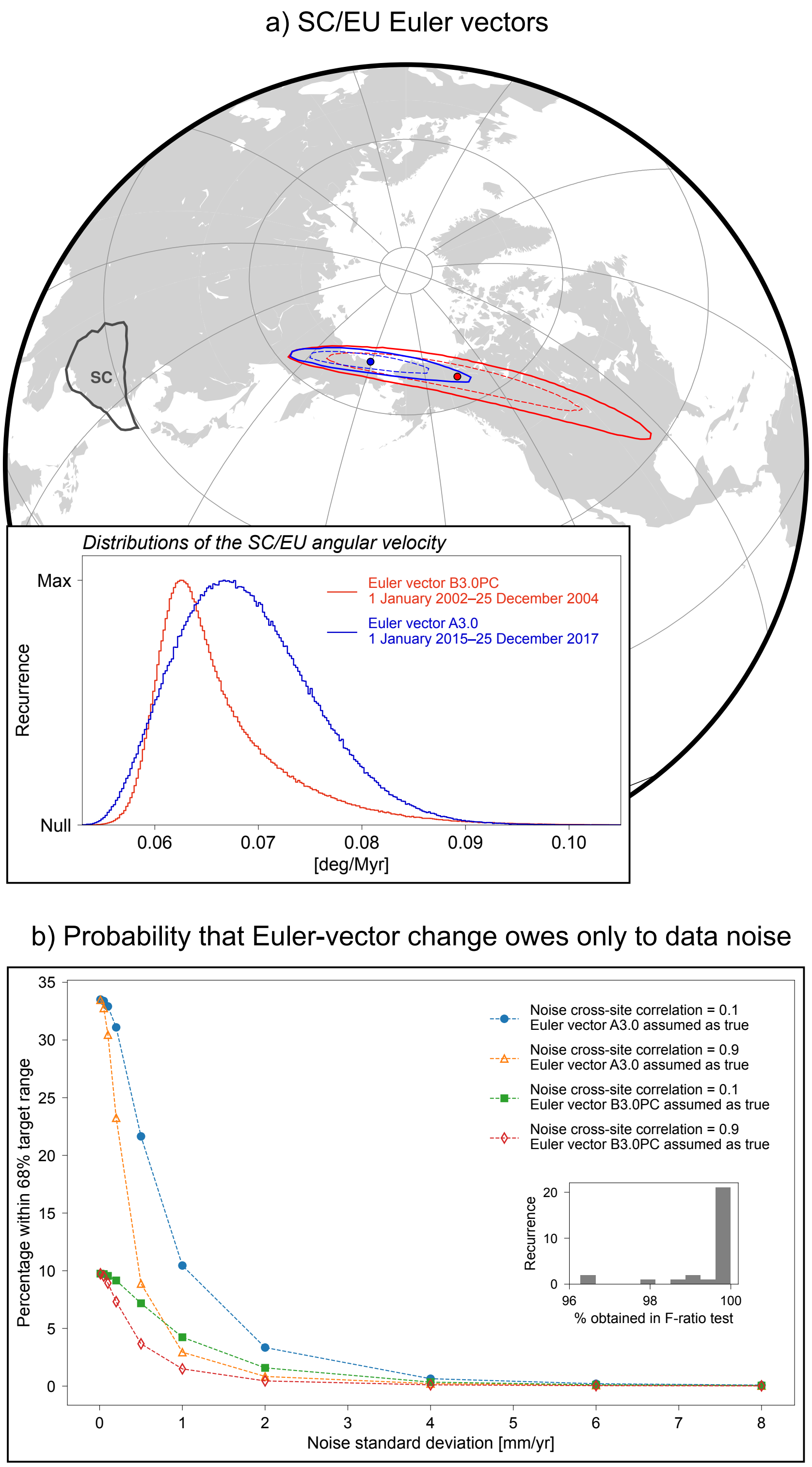


Supplementary Figure 7: Same as Fig. 4 in the main text, but for SC/EU Euler vector B3.0PC (panel a) and A3.0 (panel b) in Table 1 of the main text.

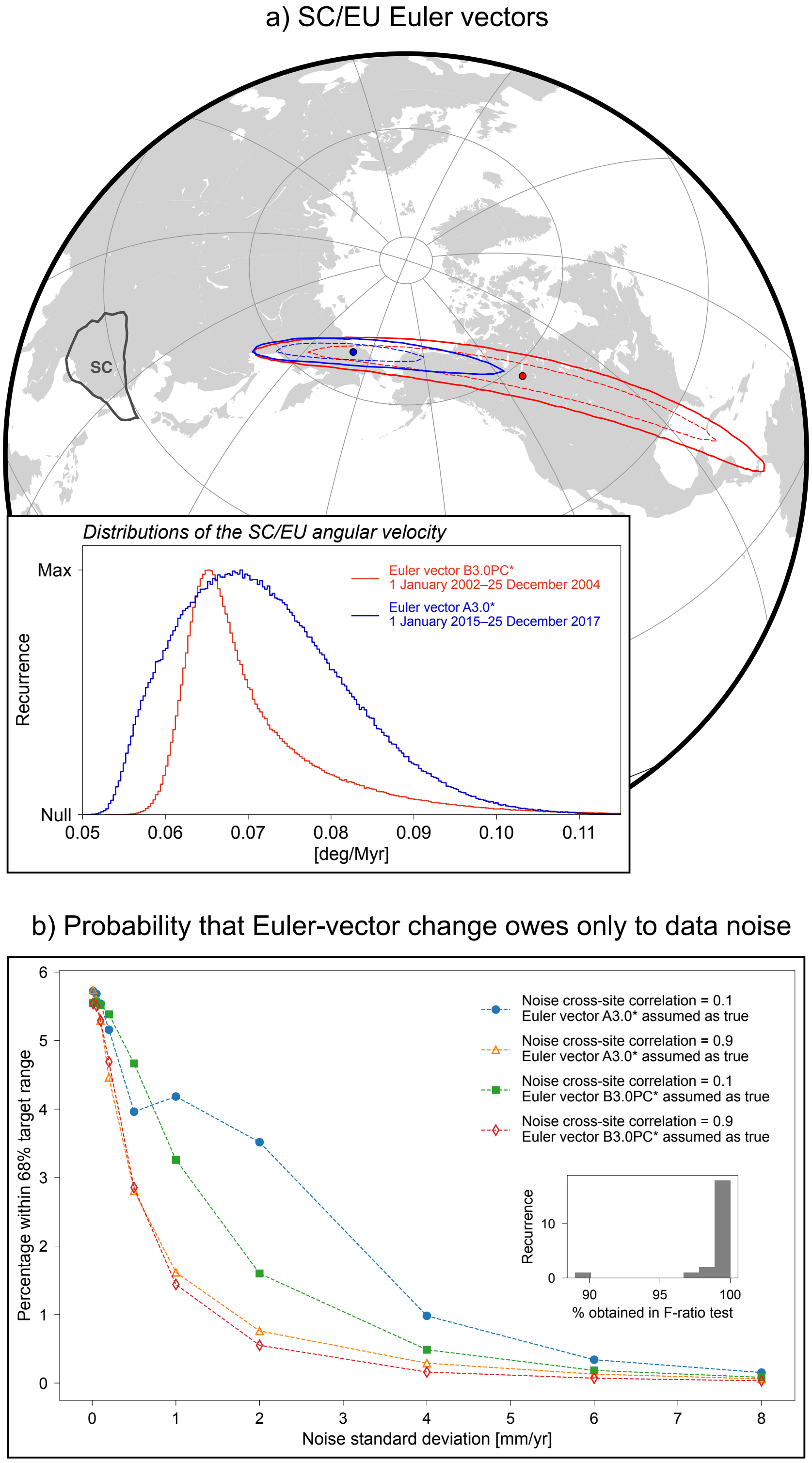


Supplementary Figure 8: Same as Fig. 4 in the main text, but for SC/EU Euler vector B3.0PC* (panel a) and A3.0* (panel b) in Table 1 of the main text.

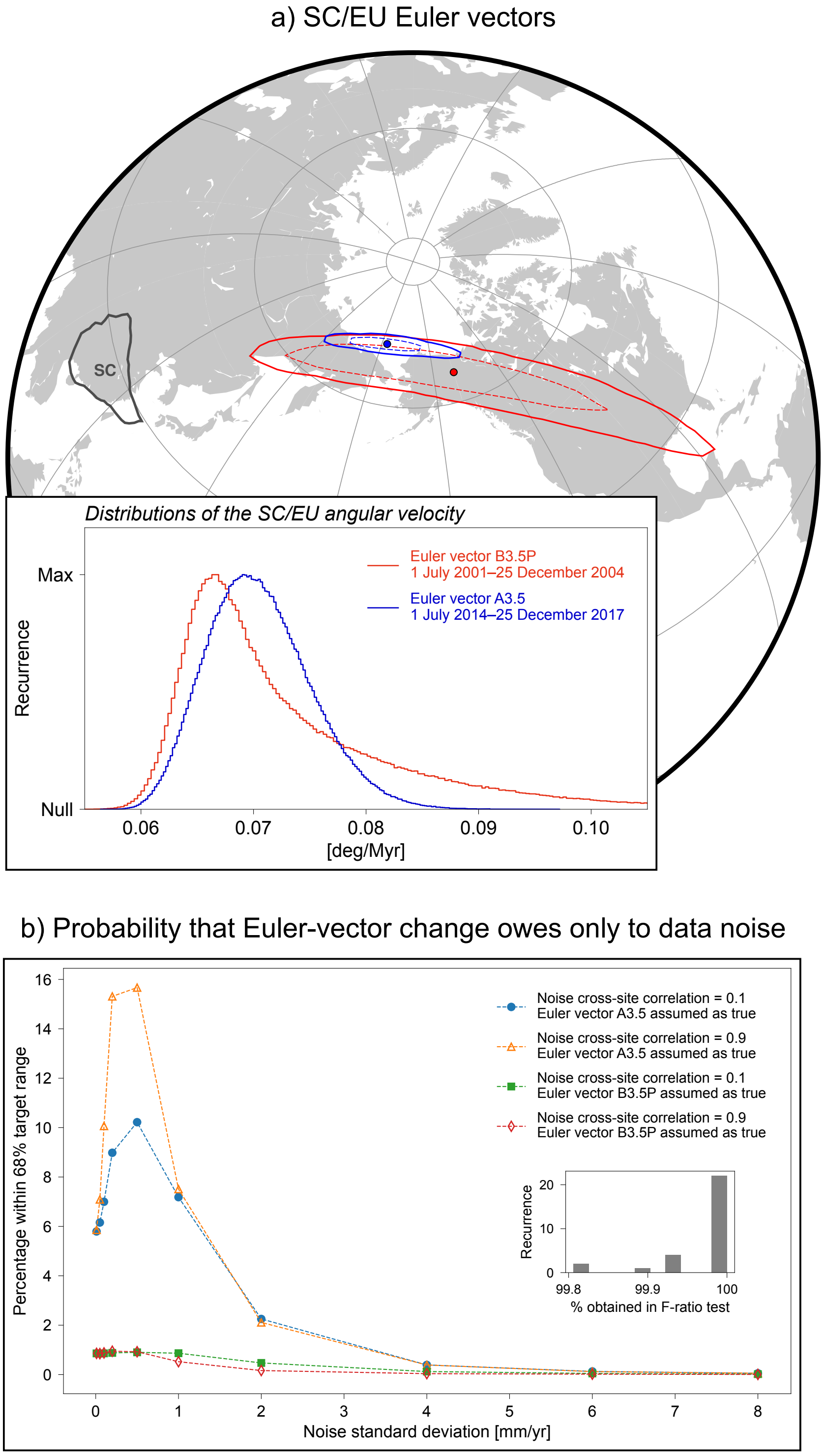


Supplementary Figure 9: Same as Fig. 4 in the main text, but for SC/EU Euler vector B3.5P (panel a) and A3.5 (panel b) in Table 1 of the main text.

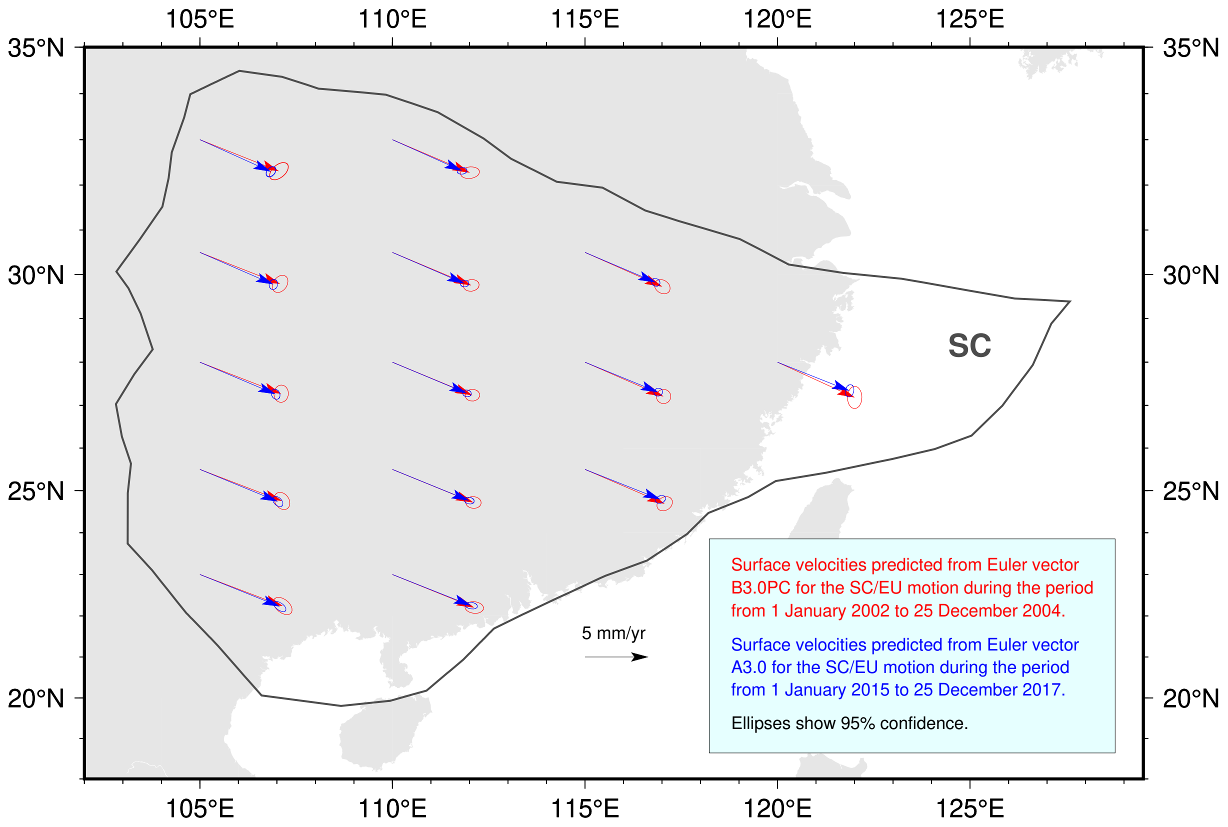


Supplementary Figure 10: Same as Fig. 5 in the main text, but for SC/EU Euler vector B3.0PC (panel a) and A3.0 (panel b) in Table 1 of the main text.

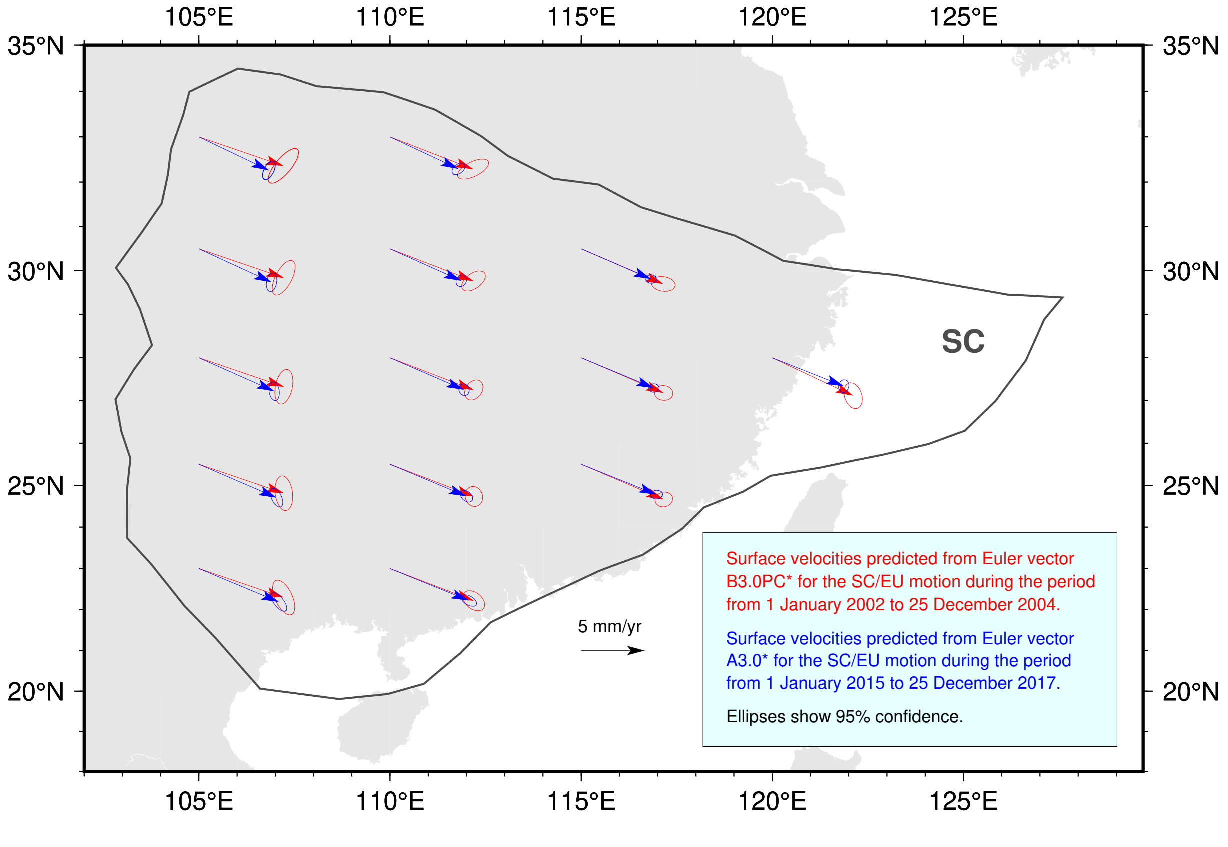


Supplementary Figure 11: Same as Fig. 5 in the main text, but for SC/EU Euler vector B3.0PC* (panel a) and A3.0* (panel b) in Table 1 of the main text.

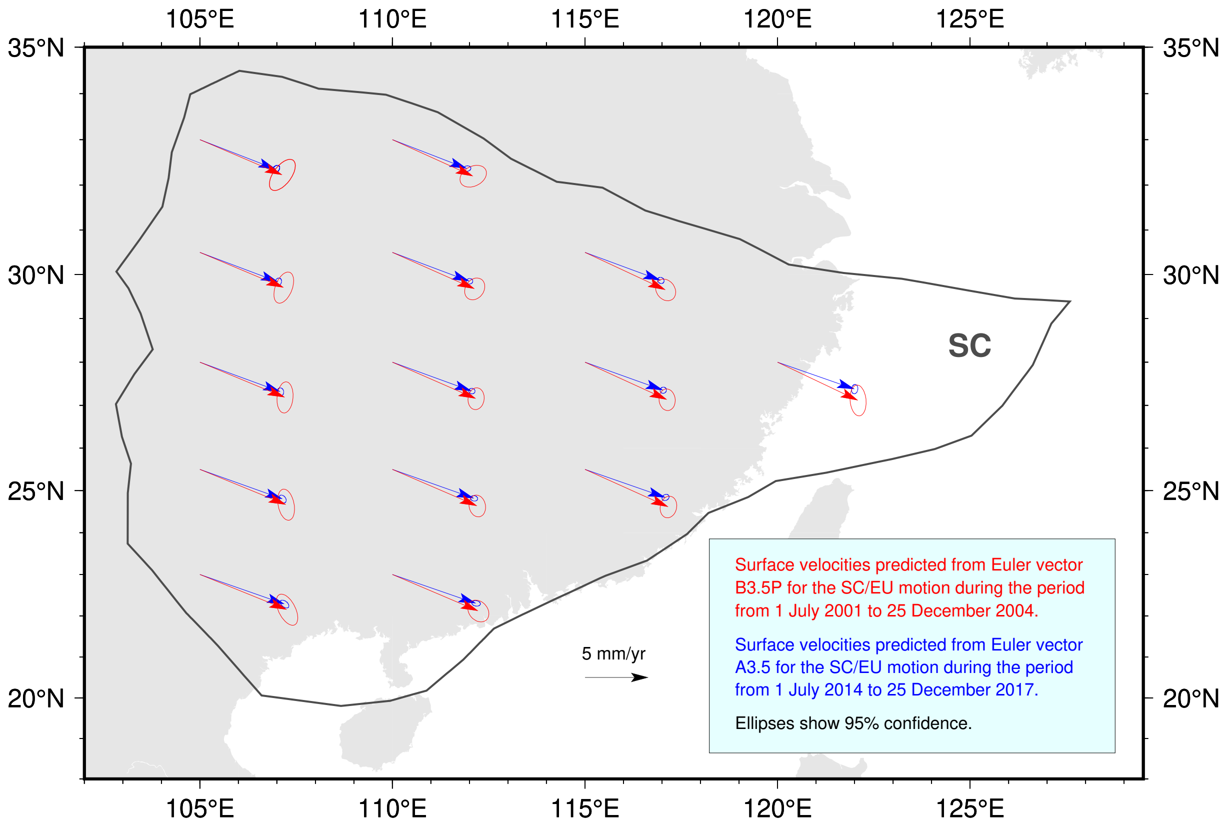


Supplementary Figure 12: Same as Fig. 5 in the main text, but for SC/EU Euler vector B3.5P (panel a) and A3.5 (panel b) in Table 1 of the main text.
